# Unraveling the Mn^2+^ substitution effect on the anisotropy control and magnetic hyperthermia of Mn_*x*_Fe_3−*x*_O_4_ nanoparticles†

**DOI:** 10.1039/d5nh00254k

**Published:** 2025-07-23

**Authors:** Oscar F. Odio, Giuseppina Tommasini, F. J. Teran, Jesus G. Ovejero, Javier Rubín, María Moros, Susel Del Sol-Fernández

**Affiliations:** a SECIHTI-Instituto Politécnico Nacional, Laboratorio Nacional de Conversión y Almacenamiento de Energía CICATA-Legaria 11500 Mexico City Mexico; b Instituto de Nanociencia y Materiales de Aragón, INMA (CSIC-Universidad de Zaragoza), C/ Pedro Cerbuna 12 50009 Zaragoza Spain m.moros@csic.es; c iMdea Nanociencia, Campus Universitario de Cantoblanco 28049 Madrid Spain; d Unidad de Nanomateriales Avanzados, iMdea Nanociencia, Unidad Asociada al CSIC Madrid Spain; e Instituto de Ciencia de Materiales de Madrid, ICMM (CSIC), Sor Juana Inés de la Cruz 3 28049 Madrid Spain; f Dept. Materials Science and Metallurgy, Escuela de Ingeniería y Arquitectura (EINA), Universidad de Zaragoza, María de Luna 3 50018 Zaragoza Spain; g Centro de Investigación Biomédica en Red de Bioingeniería, Biomateriales y Nanomedicina (CIBER-BBN) Madrid Spain; h Istituto di Science Applicate e Sistemi Intelligenti “E. Caianiello”, Consiglio Nazionale delle Richerche Via Campi Flegrei 34 Pozzuoli 80078 Italy

## Abstract

Composition is a key parameter to effectively tune the magnetic anisotropy of magnetic nanoparticles, which in turn can modulate their structural–magnetic properties and final applications. The Mn^2+^ content of manganese ferrite nanoparticles (Mn_*x*_Fe_3−*x*_O_4_) deeply impacts their structure, anisotropy, magnetism, and their heating capacity. However, a direct correlation between Mn^2+^ content, magnetic properties and heating efficiency is not yet clear. Herein, we report the synthesis of a wide range of Mn_*x*_Fe_3−*x*_O_4_ with *x* = 0.14 to 1.40, with similar polyhedral morphologies and sizes (13 to 15 nm). By varying the Mn^2+^ content (in the range of *x* = 0.0 up to 0.70), we successfully tuned the effective anisotropy while maintaining saturation magnetization nearly constant. Highest Mn^2+^ levels (*x* = 1.40) lead to structural changes and strain defects reflected in their poor saturation magnetization. Mn^2+^ substitution is not uniform, instead promotes a compositional gradient across the MNPs, with the surface layers having a higher concentration of Mn^2+^ than the core. The Mn^2+^-rich surface likely exhibits superparamagnetic (SPM) relaxation, while the core remains predominantly ferrimagnetic (FiM). Water transference results in cation leaching, promoting vacancies and changes in the local ferrite structure but with a minor impact on the magnetic properties compared with initial MNPs. We obtained the optimal Mn^2+^ content that maximizes anisotropy toward improved specific loss power (SLP) values. The Néel relaxation mechanism is warranted regarding variable composition when sizes and shapes are maintained. Our detailed analysis provides a better understanding of the effect of Mn^2+^ substitution on the heating efficiency through anisotropy modulation and straightforward guidance on optimizing MNP design for magnetic hyperthermia.

## Abstract 2

Mn^2+^ substitution creates a compositional gradient that induces core–shell magnetic behavior, enabling precise anisotropy tuning for magnetic hyperthermia.

## Graphical abstract

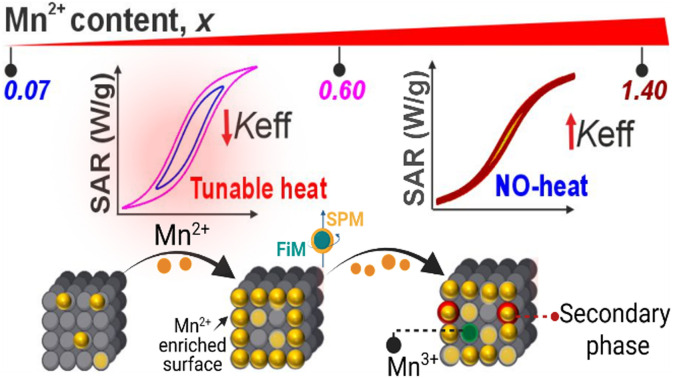

**New concepts**

We present a successful strategy to tune the heating efficiency of manganese ferrite nanoparticles (Mn_*x*_Fe_3−*x*_O_4_) by systematically varying the Mn^2+^ content, while maintaining consistent the size and shape. This approach uncovers a direct correlation between the Mn^2+^ content, effective anisotropy, and heating performance – key parameters for optimizing magnetic hyperthermia agents. A key discovery is that Mn^2+^ incorporation is not uniform – it begins on the nanoparticle surface, creating a compositional gradient. This gradient leads to superparamagnetic relaxation on the Mn-rich surface and ferrimagnetic behavior in the core. We identify an optimal Mn^2+^ content (*x* ≈ 0.60–0.70) that maximizes effective anisotropy and SLP, while preserving biocompatibility (>90% cell viability after 24 h). In contrast, high Mn^2+^ levels (*x* ≈ 1.40) lead to structural defects, Mn^3+^ species, and reduced magnetization. Despite ion leaching during water transfer *via* PMAO coating, key magnetic properties are retained. This work provides critical new insight into how compositional control – independent of morphology and size – affects magnetic relaxation and heating performance, offering a rational framework for the design of stable, efficient, and safe magnetic nanoparticles for application in nanomedicine.

## 1. Introduction

Manganese ferrite nanoparticles (Mn_*x*_Fe_3−*x*_O_4_ MNPs) have attracted considerable attention for their potential applications in magnetic hyperthermia therapy (MHT) and magnetic resonance imaging (MRI).^1^ The substitution of Fe^2+^ by Mn^2+^ in the spinel structure can significantly alter their magnetic anisotropy, saturation magnetization (*M*_S_), heating, and contrast efficiency due to changes in their structural properties, including crystal structure, crystallite size, and lattice parameter.^2–4^ While the impact of the Mn^2+^ content on the magnetic properties has been investigated in recent years using many different Mn_*x*_Fe_3−*x*_O_4_ MNPs,^5–8^ a direct correlation between the Mn^2+^ content and the magnetic properties is not yet clear. For instance, a broad range of Mn_*x*_Fe_3−*x*_O_4_ MNPs (0 ≤ *x* < 1) prepared by the co-precipitation method showed a monotone increase of lattice parameters and heating capacity as the Mn^2+^ content increased, although these results could not be exclusively attributed to the Mn^2+^ content due to the size dispersion between samples.^9^ Conversely, other authors did not find a correlation between Mn^2+^ content and magnetic properties of MNPs^7,10,11^ or reported that the net *M*_S_ values did not consistently increase with Mn^2+^ content,^12^ even when a correlation between crystallite size and Mn^2+^ content was observed. Most notably, while computational modelling revealed a positive correlation between the Mn^2+^ content and *M*_S_,^13^ experimental data showed an opposite trend, which was attributed to a reduction in both the crystallite and magnetic diameters promoted by the incorporation of Mn^2+^. Therefore, it is clear that one of the major challenges in studying how the composition of Mn_*x*_Fe_3−*x*_O_4_ MNPs impacts on the structural–magnetic properties is maintaining similar average size, shape, and monodispersity across all samples, a fact that has been frequently overlooked.

These discrepancies across reports are also reflected in the optimal conditions for effective heat dissipation under an alternating magnetic field (AMF). For instance, similar specific loss power (SLP) under an AMF has been observed in samples with markedly different composition,^14,15^ whereas samples with similar composition produced significant differences in terms of heat generation depending on their internal structural defects.^3^ The optimal Mn^2+^ content needed to efficiently dissipate heat under similar AMF conditions is not yet clear.^4,16^

Therefore, further studies are necessary to better understand how composition can affect all these properties. More importantly, in many cases, these studies are performed exclusively in organic solvents, overlooking the impact that the water transference protocols can have on the final composition and the magnetic properties, potentially leading to partial or misleading interpretations. Soriano *et al*. reported Mn^2+^ leaching, and consequently, composition changes after water transference by using meso-2,3-dimercaptosuccinic acid (DMSA) and dopamine (DOPA) ligands.^16^ This leaching could alter the crystal structure of the MNPs, significantly affecting their magnetic properties.

In this work, we present a detailed and systematic study of Mn_*x*_Fe_3−*x*_O_4_ MNPs (*x*_Empiric_ = 0.14–1.40) with similar sizes and shapes, synthesized by a one-step thermal decomposition method, with the aim of investigating Mn^2+^ substitution effects on the structural, anisotropy, magnetic behaviour and heating properties of Mn_*x*_Fe_3−*x*_O_4_ MNPs. To achieve this, we synthesized seven MNP samples by varying critical synthetic parameters, including the ratio of metallic precursors, final solvent volume, and the surfactant-to-metallic precursor ratio. A detailed investigation into the manganese oxidation state and allocation of cations among the samples revealed, for the first time, that the incorporation of manganese ions into the ferrite structure begins at the outermost layers and progressively extends toward the MNP core. We concluded that Mn^2+^ substitution is not uniform: instead, a concentration gradient is observed, with a higher Mn^2+^ fraction on the surface. The Mn^2+^-rich surface dictated the preferential magnetic relaxation of the MNPs, as seen by Mössbauer spectroscopy. Finally, we investigated the impact of a polymer coating on the final compositions of the Mn_*x*_Fe_3−*x*_O_4_ MNPs, their crystal structure and magnetic properties. This work provides valuable insights into how tailoring Mn^2+^ content can be used to effectively tune anisotropy and heating efficiency, thus advancing the understanding and optimization of MHT.

## 2. Materials and methods

### 2.1. Materials

Iron(iii) acetylacetonate (Fe(acac)_3_, 97%), manganese(ii) acetylacetonate (Mn(acac)_2_, 98%), oleic acid (OA, 90%), 1,2-hexadecanediol (technical grade, 90%), dibenzyl ether (DBE, 90%), poly(maleic anhydride-*alt*-1-octadecene) (PMAO) (MW = 30 000–50 000 Da), *N*-(3-dimethylaminopropyl)-*N*-ethylcarbodiimide hydrochloride (EDC), 4-aminophenyl β-d-glucopyranoside and 3-(4,5-dimethylthiazol-2-yl)-2,5-diphenyltetrazolium (MTT) were purchased from Sigma-Aldrich. Tetramethylrhodamine 5-(and -6)-carboxamide cadaverine (TAMRA) was purchased from Anaspec. All solvents were of analytical grade and used as received. Dulbecco's modified Eagle's medium (DMEM), fetal bovine serum (FBS), glutaMAX, and antibiotic penicillin–streptomycin (10 000 U mL^−1^) were obtained from Gibco, and 4′,6-diamidino-2-phenylindole dilactate (DAPI) and Prolong Diamond were obtained from Invitrogen. Phalloidin Alexa Fluor 488 was purchased from ThermoFisher Scientific. Glass 4-well chamber slides were obtained from Nunc™ Lab-Tek.

#### 2.1.1. Synthesis of Mn_*x*_Fe_3−*x*_O_4_ MNPs

Different ratios of the Fe(acac)_3_/Mn(acac)_2_ precursor (Fe/Mn ratio) were employed to tune the Mn_*x*_Fe_3−*x*_O_4_ composition from *x* = 0.14 to 1.4 through a one-step thermal decomposition method.^17^ The reagent amounts used for all samples are shown in Table S1 in the ESI.† Briefly, 15 mmol of the mixture of metallic precursors at different Fe/Mn ratios was dissolved in 150 mL of benzyl ether (BE) to obtain *x* = 0.14, 0.20, and 0.37 MNPs (large-scale synthesis, A Series), while 5 mmol of the metallic precursors, but the same ratios of Fe/Mn were dissolved in 50 mL of BE to obtain the MNPs with *x* = 0.40, 0.70 and 1.4 (small-scale synthesis, B Series). In the small-scale synthesis, 10 mmol of 1,2-hexadecanediol (HDD) was used, whereas in large scale synthesis, 30 mmol was employed. The oleic acid (OA)/metallic precursor ratio was slightly increased from 2.6 (on large-scale) to 3 (on small scale). The temperature and heating rate at the nucleation and growth steps were maintained invariable in both syntheses. As common steps, the mixture was mechanically stirred (100 rpm) under a smooth N_2_ flow, heated to 200 °C for 2 hours (5 °C min^−1^) and afterwards heated to 285 °C (3 °C min^−1^) for another 2 hours. Finally, the mixture was cooled down to room temperature by removing the heat source. An ethanol excess was added to the mixture, the black material was precipitated and separated with a permanent magnet. The product was then resuspended in hexane, and precipitated with ethanol four times, resulting in a brownish-black hexane dispersion that was stored at 4 °C for future use.

#### 2.1.2. Water transference

The transference into water was performed following a previously reported method with slight modifications.^18^ In brief, 225 mg of poly(maleic anhydride-*alt*-1-octadecene) (PMAO) was added to a flask containing 195 mL of chloroform and placed in an ultrasonic bath for 30 min at room temperature. Subsequently, 10 mg_Fe_ per mL in 5 mL of CHCl_3_ was added dropwise, and the mixture was sonicated for another 15 min. Afterwards, the solvent was slowly removed under vacuum (200 mbar, 40 °C). The MNPs were then resuspended in 20 mL of NaOH 0.05 M and rota-evaporated (200 mbar, 70 °C) until complete evaporation of CHCl_3_. At this point, the solution became clear as MNPs were completely transferred to water. MNPs were then filtered using 0.22 μm syringe filters. To remove the excess of unbound polymer, the MNP suspension was centrifuged at 24 000 rpm for 2 h four times and finally the MNPs were redispersed in milliQ water for further use.

#### 2.1.3. Dye functionalization

To allow *in vitro* tracking, MNPs were labelled with a fluorescent dye (tetramethylrhodamine 5-(and -6)-carboxamide cadaverine (TAMRA)).^19^ To do so, 1% of the polymer monomers were modified with TAMRA (2 mg mL^−1^) under magnetic stirring overnight in CHCl_3_ before adding the MNPs.

#### 2.1.4. Functionalization with glucose

Functionalization with glucose was performed by incubating 1 mg of Fe with 19 μmol of *N*-(3-dimethylaminopropyl)-*N*′-ethylcarbodiimide hydrochloride (EDC) and 30 μmol of 4-aminophenyl β-d-glucopyranoside in 250 μL of SSB buffer pH 9 (50 mM of boric acid and 50 mM of sodium borate). After 3 h of incubation (600 rpm, 37 °C), the ligand excess was removed by washing the MNPs with phosphate-buffered saline (PBS 1×) pH 7.4 in a centrifugal filter with a membrane of the 100 kDa molecular weight limit (Merck Millipore, Darmstadt, Germany) four times. Finally, the MNPs functionalized with glucose were filtered using syringe filters of 0.22 μm under sterile conditions and stored at 4 °C for further use.

#### 2.1.5. Structural and colloidal characterization

The MNPs’ size and morphology were evaluated by TEM using a Tecnai T20 (FEI, Netherlands) electron microscope working at an acceleration voltage of 200 kV. TEM samples were prepared by depositing 5 μL of dilute solution on a copper grid (200 mesh) and a posterior drying at ambient temperature prior to analysis. MNP size distributions were obtained by measuring more than 200 MNPs with the Image J software. High resolution transmission electron microscopy (HRTEM), scanning transmission electron microscopy (STEM), and energy-dispersive X-ray (EDX) images were obtained using a Tecnai F30 microscope with an accelerating voltage of 300 kV. Elemental mapping was performed using an Oxford EDS system in the drift corrector acquisition mode.

The crystal structure of the samples was identified by XRD powder patterns recorded using a Bruker D8 ADVANCE diffractometer working with CuK_α_ (*λ* = 1.5406 Å) radiation. The patterns were collected between 10° and 70° in the 2*θ* range. Crystallite size and lattice strain were determined using the Scherrer formula:

1 *d* = *kλ*/*β *cos(*θ*)

in which the anisotropy constant *k* = 0.94, *β* is the full width at half maximum (FWHM), and *θ* is the Bragg angle in radians. The Williamson and Hall method was used to obtain size and strain broadening by considering the peak width as a function of 2*θ* as2
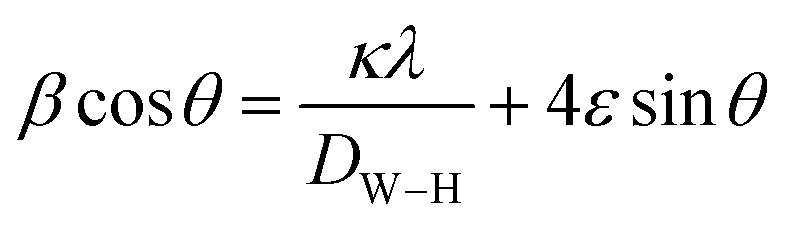
where *ε* is the strain component of the crystal lattice, and *D*_W–H_ is the average crystallite size obtained by the W–H analysis. By plotting *β* cos *θ* on the *y* axis against 4 sin *θ* on the *x* axis, and their consequent linearly fitting, we get the strain component from the slope (*ε* = slope) and the crystallite size from the intercept (*i* = *kλ*/*D*_W–H_).^20^

The elemental analysis was performed using an inductively coupled plasma optical emission spectrometry (ICP-OES) instrument (PerkinElmer mod. OPTIMA 2100 DV). Typically, 25 μL of the Mn_*x*_Fe_3−*x*_O_4_ MNPs suspension were digested in 1 mL of aqua regia in a volumetric flask at 60 °C overnight. Afterward, the flask was filled up with deionized water.

The surface chemistry was elucidated from FT-IR spectra recorded using a PerkinElmer Spectrum Two spectrometer in the range of 400–4000 cm^−1^. Samples were lyophilized 24 hours before use.

Thermogravimetric analysis (TGA) of the metal precursors was performed in a PerkinElmer TGAQ5000 instrument from 40 to 500 °C at a rate of 10 °C min^−1^ and under a N_2_ flow rate of 25 mL min^−1^. Likewise, the MNP organic contents were determined by TGA using a Universal V4.5A TA instrument; the N_2_ flow rate was 50 mL min^−1^ and the heating rate was set at 10 °C min^−1^ until a final temperature of 800 °C. MNPs in organic solvents were dried in air, while MNPs in water were lyophilized before measured.

The hydrodynamic diameters and zeta potential of the MNPs were measured using dynamic light scattering measurements (Malvern Zetasizer Nano) at room temperature. Samples were prepared at a concentration of 0.05 mg_Fe_ per mL in Milli-Q water and sonicated 10 s before measurement.

X-ray photoelectron spectra were recorded with a Kratos AXIS Supra spectrometer equipped with a monochromatic source of Al Kα (1486.7 eV) working at 120 W with a base pressure of 10^−9^ Torr; the survey spectra were recorded at a pass energy of 160 eV with a step size of 1000 meV, while the high resolution spectra were registered with a step size of 100 meV at pass energies of 20 and 40 eV for metal 2p and 3s regions, respectively. Due to the magnetic nature of the ferrite samples, the magnetic lenses were disabled, and it was operated in the electrostatic-slot mode, setting an analysis area of *ca.* 2 mm × 1 mm. The powdered samples were measured without any treatment, and the charge neutralizer mode was kept on during the measurements. All spectra were analyzed with the Thermo Avantage software package. The background was described with a Shirley-type equation. Relative atomic contents from the metal 2p region were computed from the Scofield sensitivity factors and the energy compensation factors obtained using the TPP-2M method. The high-resolution spectra in the metal 3s region were fitted with Voight profiles obtained from a mixture of Gaussian and Lorentzian functions (80/20); details concerning the calculation of the relative atomic concentrations after the fitting procedure are provided in the ESI.†

Mössbauer spectra were collected using a constant acceleration spectrometer equipped with a 5 mCi Co57/Rh source. The velocity scale was calibrated with α-Fe foil and analysed using the MossWinn 4.0 software. Isomer shift values are given with respect to α-Fe.

### 2.2. Determination of the iron concentration

The determination of the iron concentration was performed following a previously reported protocol,^21^ and the iron concentration was adjusted before glucose functionalization. In brief, 5 μL of MNPs were diluted in 45 μL of solvent (hexane or water) and digested with 100 μL of aqua regia solution (3 : 1 HCl : HNO_3_) at 60 °C for 15 min. Then, the samples were diluted up to 300 μL with miliQ water. At this point, 50 μL (in triplicate) were used for the iron quantification by mixing the digested samples with 60 μL of 0.25 M 1,2-dihydroxybenzene-3,5-disulfonic acid (Tiron). This molecule forms a coloured complex with iron, and it can be investigated by spectrophotometry at *λ* = 480 nm using a microplate spectrophotometer (BioTek Synergy H1 UV/VIS, Agilent Technologies, Santa Clara, CA, USA) and the results obtained were compared with standard calibration curve results obtained with solutions of known iron concentrations (0–1000 μg Fe per mL). This protocol was performed after each glucose functionalization.

### 2.3. Conventional calorimetry measurement

The determination of magnetic losses from MNP suspensions was performed by non-adiabatic calorimetry measurements using a nanoScale Biomagnetics D5 series device as an AC magnetic field generator. The equipment is composed of a generator with a frequency ranging from 100 to 763 kHz and a magnetic coil (magnetic field range: 0–60 mT). The suspension (1 mL) was placed into a glass vial located at the center of the magnetic induction coil inside an isolating holder. The temperature was measured with an optic fiber sensor Neoptix T1 incorporated into the equipment (measurement range – 40 to 200 °C) for 300 s. Thermal measurements of MNPs in water were performed at 2 mg_Fe+Mn_ per mL and 1 mg_Fe+Mn_ per mL for low (155 kHz) and high (763 kHz) frequency, respectively. Different magnetic field intensity was used depending on the selected frequency: *f* = 763 kHz, *H* = 3.8, 8.0, 16.8, 28.8 kA m^−1^ and for *f* = 155 kHz, *H* = 16.8, 28.8 and 44.6 kA m^−1^. The effect of viscosity (1 mL of 0%, 2% and 50% of glycerol in double distilled water) was evaluated for selected samples at *f* = 763 kHz. The temperature change was measured as a function of time (d*T*/d*t*), and the initial linear slope (*t* = 30 s) was used to evaluate the heating efficiency in terms of SLP. The SLP values were calculated according to the following expression:3
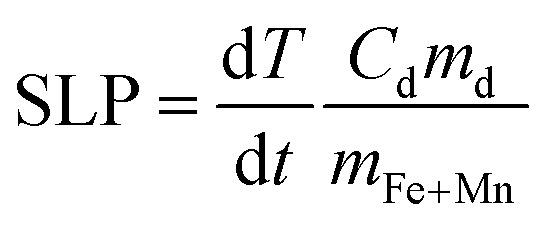
where *C*_d_ is the mass specific heat of the dispersion media, *m*_d_ is the mass dispersion, *m*_Fe+Mn_ is the iron and manganese masses related to the MNPs diluted in the dispersion and d*T*/d*t*|max is the maximal temperature slope immediately after switching *H*_AC_ on. Solvents in our experiment were water (*C*_d water_ = 4.18 J g^−1^ °C^−1^) or water with different glycerol fractions from 0 to 50%, considering *C*_d glycerol_ = 2.43 J g^−1^ °C^−1^ and for mixed water–glycerol *C*_d mix_ = 3.30 J g^−1^ °C^−1^. The sample volume employed for the experiment was 1 mL.

### 2.4. Magnetic measurements

Static conditions: magnetic characterization of MNPs under static conditions was carried out using a superconducting quantum interference device (SQUID, Quantum Design) magnetometer at distinct temperatures from 5 K up to 300 K fields up to 4000 kA m^−1^. The studied magnetic suspensions were lyophilized to get a powder, which was put into a gelatin capsule (∼5 mg per sample), and immobilized with cotton wool. Magnetization units were normalized to the organic content measured by TGA. Saturation magnetization (*M*_S_) values were calculated by extrapolating to infinite field the experimental results obtained in the high field range where the magnetization linearly increases with 1/H.

Dynamical conditions: AC magnetometry measurements of the studied magnetic suspensions (*m*_Fe+Mn_ = 5 mg) were carried out using commercial inductive magnetometers (Advance AC Hyster Series; Nanotech Solutions, Spain) at 150 kHz and 300 kHz and field intensities up to 24 kA m^−1^. Each magnetization cycle is obtained out of three repetitions, resulting in the averaged magnetization cycle, related magnetic parameters (*H*_C_, *M*_R_, Area) and magnetic losses. SAR values were determined using the expression.^22^

4 SAR = *Af*

where *A* is the AC magnetic area and *f* is the AC magnetic field frequency. Magnetization units were normalised by the magnetic mass (*i.e.* iron plus manganese masses).

### 2.5. Biological assays

#### 2.5.1. Cell culture

MIA PaCa-2 human and male pancreatic adenocarcinoma cells (ATCC® number CRL-1420) were cultured in Dulbecco's modified Eagle's medium (DMEM) supplemented with 10% fetal bovine serum, 5% l-glutamine, and 5% penicillin/streptomycin antibiotics and maintained in a humidifier incubator at 37 °C in a 5% CO_2_ atmosphere.

#### 2.5.2. *In vitro* cytotoxicity assay

MTT assay was performed to evaluate cell viability of MNP-treated cells. MIA PaCa-2 cells were seeded in 96-well plates and cultured for 24 h (5 × 10^3^ cells per well). MNPs functionalized with glucose were addedd in DMEM at various concentrations (25, 50, 100, and 150 μg Fe per mL) and incubated for 24 h at 37 °C. After treatment, cells were thoroughly washed with PBS, and 10 μL of MTT solution (5 mg mL^−1^ in PBS) in a final volume of 100 μL (DMEM w/o phenol red) was added to each well. Following 30 min of incubation at 37 °C, the plates were centrifugated at 1250 rpm for 30 min and formazan salts were dissolved with 100 μL of DMSO in agitation for 15 min at 37 °C; the resulting absorbance was determined at *λ* = 540 nm on a microplate reader (BioTek Synergy H1 Multimode Microplate Reader). Control cells without MNPs (negative control) were also analysed. Data are expressed as the percentage of cell viability normalized for the control condition and are the results of three independent biological replicates.

#### 2.5.3. Internalization by fluorescence microscopy

MNP internalization was studied by fluorescence microscopy (using MNPs modified with TAMRA fluorophore) and ICP-OES. Fluorescence microscopy: to perform fluorescence microscopy analysis, 5 × 10^4^ cells were seeded on a glass 4-well chamber slide. After 24 h of culturing, cells were treated with 100 μg Fe per mL of MNPs functionalized with glucose and incubated at 37 °C (5% CO_2_) for 24 h. Noninternalized MNPs were removed by washing with PBS thrice. Cells were fixed with 4% of paraformaldehyde for 15 min at room temperature and washed twice with cold PBS. After that, F-actin staining was performed using phalloidin AlexaFluor 488 following manufacturer's instructions. For nuclei labelling, cells were incubated 10 min at room temperature with 0.6 μM of DAPI. Finally, the slide was mounted using ProLong mountant medium. Fluorescence microscopy images were obtained using an inverted microscope (Nikon Eclipse Ti-E, Amsterdam, The Netherlands).

### 2.6. Statistical analysis

All data are presented as mean ± standard deviation (SD). One-way analysis of variance (ANOVA) and the Tukey post-hoc test were applied to calculate the differences between the values. Values of *p* < 0.05 were considered statistically significant.

## 3. Results and discussion

### 3.1. Design of a library of Mn_*x*_Fe_3−*x*_O_4_ MNPs. Structural changes as a result of manganese substitution

Several Mn_*x*_Fe_3−*x*_O_4_ MNPs with different Mn^2+^ content but similar sizes and shapes were synthesized by a one-step thermal decomposition method, modifying different parameters such as the metallic precursors ratio (Fe(acac)_3_/Mn(acac)_2_), the surfactant/precursors ratio (OA/metallic precursors) and/or the final solvent volume (see Table S1 in the ESI†). In all cases, the remaining synthetic parameters, that is, the heating rate, the reaction time, and the temperatures during the nucleation and growth steps, were maintained constant. Benzyl ether (BzE) was selected as a passive solvent due to its lack of hydroxyl groups capable of iron chelation, thereby minimizing a direct influence on the MNPs’ size and shape.^23^

To elucidate the influence of the surfactant concentration on the overall composition, two Mn_*x*_Fe_3−*x*_O_4_ MNPs were synthesized using the same Fe(acac)_3_/Mn(acac)_2_ ratio (6.5) and a final solvent volume (50 mL), while slightly varying the OA/metallic precursor ratio from 2.6 to 3 (see Table S2 in the ESI†). The empirical *x* value, determined by ICP-OES, remained unchanged for both samples (*x*_Empiric_ = 0.18), indicating that under these synthetic conditions Mn^2+^ incorporation was insensitive to slight variations in the amount of surfactant. However, the sample synthesized using lower OA/metallic precursor ratios exhibited more irregular shapes (Fig. S1 in the ESI†). A plausible explanation is that limited OA availability leads to a preferential growth of the initial seeds along certain crystal directions (particularly those normal to the facets {111} and {110}, which are poorly stabilized by the OA, resulting in more irregular shapes. In contrast, sufficient OA during the nanocrystal growth ensures well-capped and stabilized crystal facets, leading to MNPs with controlled faceted shapes.^24,25^

The overall composition of the MNPs was then varied by changing the metallic precursor ratio and final solvent volume. Two series of Mn_*x*_Fe_3−*x*_O_4_ MNPs were synthesized: A Series (large-scale synthesis), where the MNPs were prepared in 150 mL of BzE, and B Series (small-scale synthesis) obtained using 50 mL of BzE. The Mn^2+^ content in both series was adjusted by changing the initial Fe(acac)_3_/Mn(acac)_2_ ratios, corresponding to theoretical *x* values of 0.40, 0.75, and 1.0 (Table S1 in the ESI†). In all cases, Mn^2+^ incorporated into the final MNPs was much lower than the theoretical values, based on the amount of Mn^2+^ added as initial precursor. This effect has already been described and is likely due to various factors, such as differences in the decomposition temperatures of the two precursors (most of the Mn(acac)_2_ decomposes at temperature of approximately 60 °C higher than that of Fe(acac)_3_, see Fig. S6 left panel in the ESI†) and/or the ionic radii differences between Mn^2+^ and Fe^2+^.^16,26^ However, this effect was not observed in other studies when using the same metallic precursors^3,13^ or oleates.^2^ Nevertheless, in the latter, octadecene was used as the synthetic solvent instead of BzE, making a direct comparison difficult. Moreover, it should be noted that in both series of MNP samples (A and B), the higher the molar concentration of the Mn^2+^ precursor used in the synthesis, the higher the Mn content in the final ferrite stoichiometry (Table 1). This tendency, determined by ICP-OES, was further confirmed by EDX for samples belonging to A Series (Fig. S2 in the ESI†).

**Table 1:** Summary of Fe(acac)_3_/Mn(acac)_2_ molar ratios employed in the synthesis and theoretical (*x*_Theo_) and empirical (*x*_Empiric_) stoichiometries of the Mn_*x*_Fe_3−*x*_O_4_ ferrites. The average sizes were determined by TEM (*D*_TEM_). Peak position at 311 reflection and crystallite sizes determined by XRD using the Sherrer equation (*D*_XRD_) or Williamson–Hall method (*D*_W–H_)

Samples	Fe/Mn precursors’ molar ratio in synthesis	*x*_Theo_ value	*x*_Empiric_ value	Measured formula	*D*_TEM_ (nm) ± SD	(311) peak pos. (2*θ*°)	*D*_XRD_ a (nm) ± SD	*D*_W–H_ b (nm) ± SD
A Series (150 mL)	6.5	0.40	0.14	Mn_0.14_Fe_2.86_O_4_	14 ± 3	35.43	12 ± 1	12 ± 1
	3	0.75	0.23	Mn_0.23_Fe_2.77_O_4_	13 ± 3	35.40	13 ± 1	18 ± 2
	2	1.0	0.37	Mn_0.37_Fe_2.63_O_4_	15 ± 2	35.39	14 ± 2	15 ± 1

B Series (50 mL)	6.5	0.40	0.18	Mn_0.18_Fe_2.82_O_4_	13 ± 2	35.42	14 ± 1	15 ± 1
	3	0.75	0.47	Mn_0.47_Fe_2.53_O_4_	15 ± 1	35.39	13 ± 1	16 ± 2
	2	1.0	0.70	Mn_0.70_Fe_2.30_O_4_	14 ± 2	35.35	13 ± 1	16 ± 1
	1	1.5	1.40	Mn_1.40_Fe_1.60_O_4_	14 ± 1	35.26	13 ± 1	15 ± 2

a Calculated using the Scherrer method.

b Calculated using the Williamson–Hall method.

Additionally, the solvent volume influenced the incorporation of Mn^2+^ into the spinel structure (Fig. S3 in the ESI†). When the same Fe(acac)_3_/Mn(acac)_2_ ratio was used, the amount of Mn^2+^ incorporated into the spinel structure was consistently higher in the B Series (smaller volume of solvent) than in A Series (larger solvent volume) (Table 1). One hypothesis is that the use of a smaller solvent volume facilitates higher and more uniform temperatures throughout the mixture, improving the decomposition of the Mn(acac)_2_ precursor and thereby reducing the critical gap between the decomposition of metallic precursors and the saturation limit for particle nucleation.^27^ To further increase the Mn^2+^ content in the final MNPs, an additional synthesis was performed only for Serie B, as was the one incorporating more Mn^2+^ in the final structure (*x*_Theo_ = 1.5, Mn_1.50_Fe_1.50_O_4_). The empirical *x* value was similar to the theoretical one (*x*_Empiric_ = 1.40, Mn_1.40_Fe_1.60_O_4_).

**Fig. 1:**
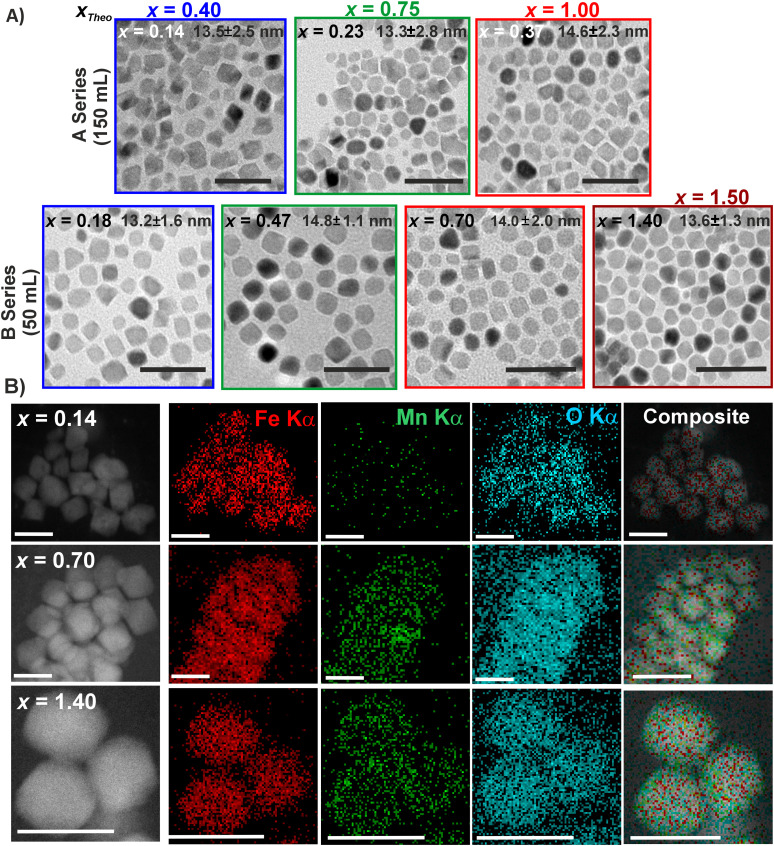
(A) TEM images of Mn_*x*_Fe_3−*x*_O_4_ MNPs with varying Mn^2+^ content (*x*_Empiric_ = 0.14 to 1.40). The samples with the initial Fe/Mn precursor amount of *x*_Theo_ = 0.40, 0.75, 1.0, and 1.50 are highlighted with blue, green, red, and brown boxes, respectively. Inset: Empirical *x* value for each sample determined by ICP-OES along with their respective size diameters. Scale bar: 50 nm. (B) STEM-EDX mapping images of Mn_*x*_Fe_3−*x*_O_4_ MNPs with the lowest, medium, and highest content of Mn^2+^ (*x*_Empiric_ = 0.14, 0.70, 1.40). Scale bar: 50 nm (*x*_Empiric_ = 0.14 and 0.70), 20 nm (*x*_Empiric_ = 1.40).

As shown in Fig. 1A and Table 1, all the synthesis yielded MNPs with similar polyhedral morphologies and sizes ranging from 13 to 15 nm, regardless of the metallic precursor's ratio or the solvent volume used. These results are in good agreement with previous works using the same precursors but different synthetic methods.^13,16,28^ Controlling the particle size and shape is crucial to isolate and accurately assess the influence of composition on the anisotropy and magnetic heating, while minimizing the influence of these parameters.

To investigate whether the tuned compositions could result in a single or core/shell MNP structure as previously reported,^29,30^ we carried out STEM-energy-dispersive X-ray spectroscopy analysis of selected Mn_*x*_Fe_3−*x*_O_4_ MNPs with the lowest, medium, and highest Mn^2+^ content (*x*_Empiric_ = 0.14, 0.70 and 1.40 respectively) (Fig. 1B). The chemical maps showed the presence of Mn and Fe signals along the magnetic cores and the absence of a marked core–shell structure, thus, confirming the successful synthesis of a unique Mn_*x*_Fe_3−*x*_O_4_ core in agreement with several reports.^2,3,16^

**Fig. 2:**
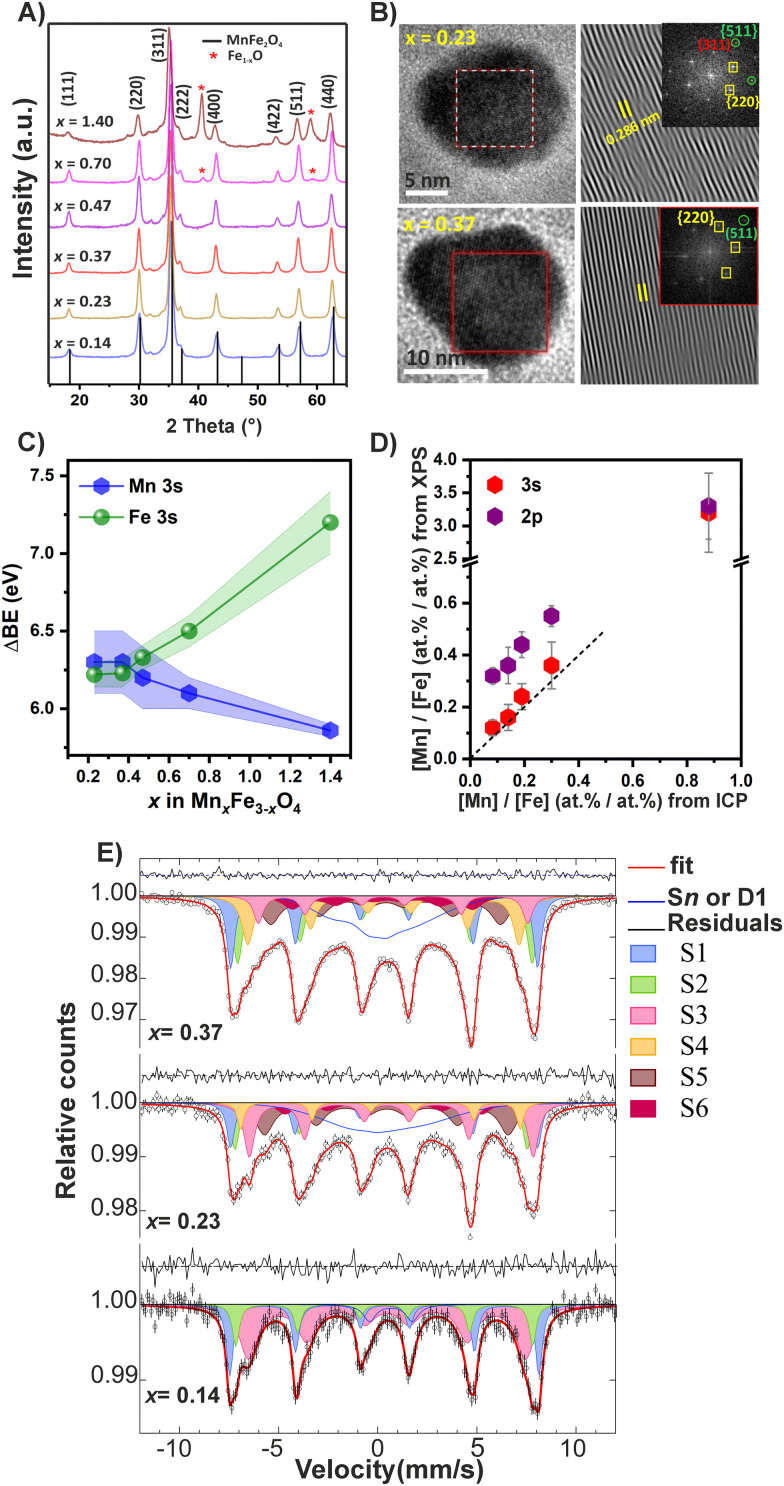
(A) Evolution of the XRD pattern of the full set of Mn_*x*_Fe_3−*x*_O_4_ MNPs with *x* = 0.14 up to 1.40 with the corresponding Bragg positions of MnFe_2_O_4_ reference (black lines) and Fe_1−*x*_O (red asterisk). (B) HRTEM images of samples with *x*_Empiric_ = 0.23 and 0.37 and their FFT and inverse FFT of the plane (220). (C) Energy splitting of the Mn 3s and Fe 3s doublets as a function of the ferrite stoichiometry computed from XPS. (D) Mn/Fe atomic ratios computed from the 2p and 3s XPS signals *vs.* the compositional data obtained from ICP, for the samples with ferrite stoichiometries of *x*_Empiric_ = 0.23, 0.37, 0.47 and 1.40. (E) Mössbauer spectra of *x*_Empiric_ = 0.14, 0.23 and 0.37 MNPs.

For further analysis, we selected samples with increasing Mn^2+^ content, that is, *x*_Empiric_ = 0.14, 0.23, 0.37, 0.47, 0.70, and 1.40. Fig. 2A shows the evolution of the XRD patterns of these MNPs together with the positions of the peaks corresponding to the MnFe_2_O_4_ spinel-type phase (PDF file 96-230-0619 in the ICDD powder diffraction file database). Interestingly, the presence of additional reflections (denoted with an asterisk) corresponding to the Fe_1−*x*_O phase (PDF file 96-101-1199) appeared for the samples with Mn^2+^ content *x* ≥ 0.70. The coexistence of both magnetic phases can have a high impact on the structural, magnetic, and heating properties of ferrite MNPs, as previously reported.^3,31,32^ As expected, we found a gradual shift toward lower angles while increasing the Mn^2+^ content from 35.43 (*x*_Empiric_ = 0.14) to 35.26 (*x*_Empiric_ = 1.40), taking as reference the reflection (311). This corresponds to an increase in the cubic unit cell parameter due to the substitution of smaller host ions (0.65 Å for Fe^3+^ and 0.78 Å for Fe^2+^) by a larger Mn^2+^ (0.83 Å),^33^ and to the appearance of strains inside the spinel structure.^2,20^ To further confirm this hypothesis, we calculated the strain (*ε*) component inside the spinel structure following the Williamson–Hall (W–H) method^33^ using XRD patterns and measuring the lattice distance for selected samples by HRTEM.^33^ As shown in the HRTEM images (Fig. 2B), the inverse FFT of the (220) plane distance increased from 0.286 nm (*x*_Empiric_ = 0.23) to 0.290 nm (*x*_Empiric_ = 0.37), in agreement with the XRD patterns. On the other hand, from the fitting of the W–H plot, we found that when the Mn^2+^ content increased from *x*_Empiric_ = 0.14 up to 1.40, the lattice strain increased from 0.20 × 10^−3^ to 2.12 × 10^−3^ (Table S3 in the ESI†), indicating a larger lattice spacing due to the introduction of Mn^2+^. This ultimately leads to the appearance of uniform tensile strain,^20^ which corresponds to the left shift shown in the XRD patterns (Fig. 2A). Although the lattice defects varied among the samples, the effect was more prominent for the sample with the highest content of Mn^2+^ (Mn_1.40_Fe_1.60_O_4_).

The average crystallite sizes calculated using Scherrer's formula (*D*_XRD_) and the W–H method (*D*_W–H_) ranged from 12 nm to 14 nm and from 12 nm to 18 nm, respectively (Table 1). In all cases, the mean MNP diameters obtained from TEM (*D*_TEM_) matched relatively well with the crystal sizes determined by XRD using both methods, indicating a single-crystal structure. No clear correlation between the Mn^2+^ content and the crystallite size was observed. Similar findings have been reported for Mn_*x*_Fe_3−*x*_O_4_ nanoparticles with homogeneous characteristics (∼6 nm in size, uniform shape, and oleic acid coating) in the range of *x* = 0.11 to 0.49.^12^ In contrast, other works have reported an increase of the crystallite size for Mn_*x*_Fe_3−*x*_O_4_ MNPs obtained by a similar synthetic procedure but using 1-octadecene as a solvent, or by co-precipitation in water using metallic chloride precursors.^4,13^ In contrast, a decrease in the crystallite size from 16.7 nm (*x* = 0) to 10.2 nm (*x* = 1) has been observed for Mn-doped CoFe_2_O_4_ ferrites.^34^ These discrepancies may be attributed to structural defects frequently associated with the increase of Mn^2+^ in the spinel structure, which could cause contraction of the crystal structure. In our case, the use of BzE as the solvent, rather than more reducing solvents like 1-octadecene, likely helped minimize the formation of such defects.^3^ Therefore, even when an increase in the Mn^2+^ content may have introduced some strain defects, these were insufficient to impact the final crystallite size of our MNPs.

### 3.2. Manganese substitution induces changes in the oxidation state, relocation of cations, and sample heterogeneity

To shed light on the oxidation state of the cations in the samples under study, we analyzed the photoelectron spectral region corresponding to the Mn 3s and Fe 3s region using X-ray photoelectron spectroscopy (XPS). The fitting results for the Mn_*x*_Fe_3−*x*_O_4_ MNPs with *x*_Empiric_ = 0.23, 0.37, 0.47, 0.70, and 1.40 are shown in Fig. S5 and summarized in Table S4 of the ESI;† also, a detailed explanation of the spectral features found in this region is provided in Section S1 of the ESI.†

All spectra were successfully fitted with two sets of doublets and a minor contribution at higher binding energy (BE). The doublet with the lower BEs (80–90 eV) corresponds to Mn cations, and the one with higher BEs (90–100 eV) is attributed to Fe cations. The occurrence of these doublets is primarily due to the multiplet splitting of the ionic final-state configuration 3s^1^3d^*n*^, arising from the exchange interactions between the remaining 3s core-electron and the unpaired electrons in the valence 3d shell of the transition metals.^35–37^ This interaction gives rise to high spin (lower BE) and low spin (higher BE) final-state configurations, where the electron spins in the 3s and 3d shells are coupled either parallel or anti-parallel, respectively. Since the magnitude of the resulting energy splitting (ΔBE) is proportional to the total spin density of the 3d shell, this value has been used to identify the oxidation state of the ion,^38,39^ especially for compounds with high degree of ionicity. In addition, as the ΔBE is also proportional to the exchange integral between the 3s and 3d shells, it could be sensitive to covalent and ligand field effects.^40,41^

Fig. 2C shows the behavior of the computed ΔBE values after spectral fitting. Remarkably, a close correlation between the energy splittings of both Mn 3s and Fe 3s doublets is apparent. For the samples with *x*_Empiric_ ≤ 0.37, the splitting of the Mn 3s doublet is higher than 6.2 eV, which strongly suggests that the manganese ions are preferentially in the divalent state, with no evidence of oxidized species.^39,42^ These values are consistent with the results reported elsewhere for several manganese ferrites.^43,44^ At these low Mn^2+^ levels (*x*_Empiric_ ≤ 0.37), the recorded values of ΔBE for the Fe 3s doublet around 6.2–6.3 eV suggest the coexistence of Fe^2+^ and Fe^3+^ cations due to the partial substitution of Mn^2+^ in the ferrite structure.^36,37,40,41^ It is worth noting that, as the Mn^2+^ content in the ferrites increases (*e.g.*, in samples with *x*_Empiric_ = 0.47 and 0.70), a gradual decrease in ΔBE of the Mn 3s doublet is observed. Such behavior could be related to either the appearance of a small fraction of Mn in a higher oxidation state (*x*_Empiric_ ≥ 0.70) or a less ionic environment of the Mn^2+^ ions in the spinel structure,^45,46^ which could suggest: (i) a gradual occupancy of tetrahedral sites by Mn^2+^ or (ii) a higher probability of –Mn^2+^–O–Mn^2+^– sequences at the expense of –Mn^2+^–O–Fe^3+^– sequences. At the same time, the splitting of the Fe 3s doublet increases, which is consistent with the progressive substitution of Fe^2+^ cations by Mn^2+^ in the spinel structure. In the case of the sample with the highest Mn^2+^ content (*x*_Empiric_ = 1.40), the further reduction of the Mn 3s ΔBE to 5.9 eV may indicate the coexistence of a Mn^3+^ fraction forming oxidized species. Meanwhile, the high value for the Fe 3s ΔBE (7.2 eV) indicates the predominant presence of Fe^3+^ cations inside the oxygen lattice consistent with a highly ionic environment.^36,40,41,47^ This observation suggests a preferential occupancy of the octahedral sites (B) by Fe^3+^.^45,46^ Additionally, the likely absence of Fe^2+^ ions is consistent with a massive substitution of Mn in the ferrite structure.

The recorded XPS spectra also provided a conclusive clue on the heterogeneity of the samples by analyzing the 2p and 3s signals of the cations. We have computed the Mn/Fe atomic ratios from the spectral fittings of the 3s region (Section S1 in the ESI†) and the direct integration of the corresponding 2p high-resolution spectra (see Fig. S4 in the ESI†). Using the TPP-2 formula previously reported,^48^ it is possible to estimate the inelastic mean free path (IMFP) of the photoelectrons coming from both spectral regions, which is a function of the photoelectron kinetic energy. For the Mn and Fe 3s photoelectrons with kinetic energy around 1400 eV, the IMFP is roughly 2.1 nm; in the case of the Mn and Fe 2p photoelectrons with kinetic energy about 800 eV, the IMFP is roughly 1.4 nm. Therefore, the information depth (3 times the IMFP) derived from the 2p and 3s signals covers the outermost 4 nm (2p) and the topmost 6 nm (3s) of the ferrite MNPs, respectively. The comparison between the computed Mn/Fe atomic ratios is displayed graphically in Fig. 2D, where both data sets derived from XPS are plotted against the Mn/Fe ratios obtained by ICP quantification. The dashed line with a unitary slope represents the ideal case where the surface composition computed by XPS agrees with the overall composition of the MNPs (ICP). From the graph, it is apparent that for the samples with *x*_Empiric_ ≤ 1.40, the estimated cation composition along the outermost 6 nm of the MNP (3s) is similar to the composition from the ICP data, which is consistent with the estimated average MNP diameters of 13–15 nm from TEM and DRX measurements. The fact that the 3s data are slightly biased toward higher Mn content in all samples could suggest that the Mn ions entering the ferrite structure do not reach the very core of the MNPs. On the other hand, by comparing the data sets from the 2p and 3s signals, it is noticeable that for *x*_Empiric_ ≤ 1.40 the Mn/Fe atomic ratio is systematically higher in the first 4 nm than if it is computed extending the probed depth for other 2 nm inside the MNP core. In other words, the nanoparticle's surface is enriched in the Mn^2+^ content with respect to the deeper zones near the core. This fact could indicate that the incorporation of Mn into the ferrite structure starts in the most superficial layers and then extends toward the interior of the MNPs.

Previous works^50–52^ on doped ferrites have also reported this surface enrichment of the doping element, which seems to be related to differing onsets of the nucleation process: when the nucleation step of the doping-containing monomers occurs late compared to the nucleation of the matrix-containing monomers, the doping element is initially out of the core of the resulting particle, and can develop a gradient concentration as a function of its diffusivity across the particle. This phenomenon is triggered by the aforementioned 60 °C difference in the decomposition temperature between Mn^2+^ and Fe^3+^ acetylacetonates, which produces monomers at different stages of the reactions that nucleate inhomogeneously. A thorough understanding of the magnitude of Mn gradient along the nanoparticle structure as a function of ferrite stoichiometry requires more in-depth studies using STEM–EELS and XPS combined with Ar ion cluster sputtering, which will be the subject of future work.

### 3.3. Mn substitution and heterogeneity in the Mn content along the MNPs dictate the predominant magnetic relaxation regime

To elucidate the impact of compositional heterogeneity and Mn substitution on the magnetic behaviour, we recorded Mössbauer spectra at room temperature. Fig. 2E shows the corresponding spectra for samples with *x*_Empiric_ = 0.14, 0.23 and 0.37. The general aspect of the spectra is different from that of bulk Mn_*x*_Fe_3−*x*_O_4_^49^ and similar to that of nanostructured ferrite MNPs.^50^ At first sight, it is apparent that the samples display magnetic order, owing to the hyperfine peaks that dominate the profiles; in addition, the occurrence of a broad central background, especially for the samples with higher Mn^2+^ content, could indicate the presence of partially superparamagnetic (SPM) components.^50^ The simultaneous presence of both features shows that the MNPs cannot be simply described as homogeneous with a constant Mn^2+^ concentration, and thus the same magnetic behaviour throughout the particle or in all the particles. Therefore, we tried an analysis intending to separate the ferrimagnetic (FiM) behaviour from the SPM-like one by considering: (1) sextets with hyperfine fields (HF) above a threshold value of 40 T (FiM portion contribution),^50–52^ and (2) sextets with lower HF values and poorly structured central contributions (fraction with SPM-like behaviour). These last features could include either a broad singlet, or a quadrupolar doublet, or a Voigt-based Gaussian distribution of HFs and isomer shifts (IS);^53^ this type of distribution can provide flexibility to the fits to simulate more complex structures at the centre of the spectrum.

The results of the fits are shown in Fig. 2E and summarized in Table S10 of the ESI.† For the sample with *x*_Empiric_ = 0.14, the spectrum was successfully fitted with three hyperfine sextets and a small quadrupolar doublet. The two sextets with higher HFs and IS values around 0.3 mm s^−1^ are attributed to Fe^3+^ ions; the one displaying the largest HF (S1) corresponds to the cations that occupy A sites, while the other sextet (S2) is assigned to the cations occupying the B sites. The third sextet (S3) with a lower HF (*ca.* 43 T) and an IS near 0.5 mm s^−1^ can be attributed to the fictitious intermediate state Fe^2.5+^ due to the fast electron hopping between adjacent Fe^2+^ and Fe^3+^ cations along the B sites of the cubic spinel, above the Verwey temperature. As can be seen, the emergence of S2 is due to the imbalance between Fe^2+^ and Fe^3+^ cations due to the partial substitution of Fe^2+^ by Mn^2+^ in the ferrite structure. For the other two measured samples (*x*_Empiric_ = 0.23 and 0.37), additional sextets (S4–S6) were required. S4 is attributed to Fe^2.5+^ states where the Fe^2+^ and Fe^3+^ cations have different local environments with respect to those in S3 due to the emergence of inequivalent sites for Fe cations, *i.e.*, the gradual incorporation of Mn^2+^ ions modifies the nearest-neighbor surroundings of Fe cations along the ferrite lattice. In contrast, S5 and S6 have large peak widths and HFs below the threshold value (40 T) for FiM behavior, indicating that these contributions encompass a fraction of Fe^3+^ and Fe^2.5+^ sites where the effective magnetic moment is no longer in a blocked FiM state, but it begins to fluctuate at a frequency that is on the order of the inverse of the Mössbauer characteristic time.

The rest of the spectral area contains only a broad quadrupolar doublet D1 (for the sample with *x*_Empiric_ = 0.14), or a distribution of HF values S*n* (for the samples with *x*_Empiric_ = 0.23 and 0.37); such distribution is similar in both samples, with IS values centered at 0.3 mm s^−1^, pointing to a dominant contribution from Fe^3+^ cations and consistent with high Mn^2+^ substitution. The relative area of D1 is small in the *x*_Empiric_ = 0.14 sample (below 6%), while for the samples with higher Mn^2+^ concentration the overall contribution from S*n* plus S5 and S6 sextets drastically increases above 40%. As was mentioned before, these spectral features indicate the occurrence of SPM relaxation, which can be the result of either small MNPs or MNPs with low magnetic anisotropy. Since the three samples exhibit similar size distributions, it is likely that the differences between them mostly arise from anisotropy effects across the particles. Indeed, the fact that the SPM contribution is more important for the samples with higher Mn^2+^ content strongly suggests that substitution of Fe^2+^ by Mn^2+^ in the ferrite lattice induces a decrease of the superexchange interactions,^51^ that is reflected in lower values of the magnetic anisotropy^54,55^ (see the next section). Moreover, since the XPS results point to a gradient of concentration with a large [Mn]/[Fe] molar fraction on the outermost part of the MNPs, it is likely that such SPM relaxation occurs preferentially on the surface of the MNPs. Hence, it can be inferred that sextets S1–S4 are associated with the inner part of the MNPs, *i.e.* the MNP cores exhibit FiM behavior at room temperature. Such a finding is further confirmed by estimating the ferrite stoichiometry from the relative area of these four contributions: the [Mn]/[Fe] molar ratio is 0.08, 0.13 and 0.20 for *x*_Empiric_ = 0.14, 0.23 and 0.37, respectively; these values are very close to those obtained from the 3s XPS data (*cf.*Fig. 2D), which provide information on the inner part of the MNPs, as has been pointed out.

### 3.4. Manganese substitution controls anisotropy and soft ferrite transition

**Fig. 3:**
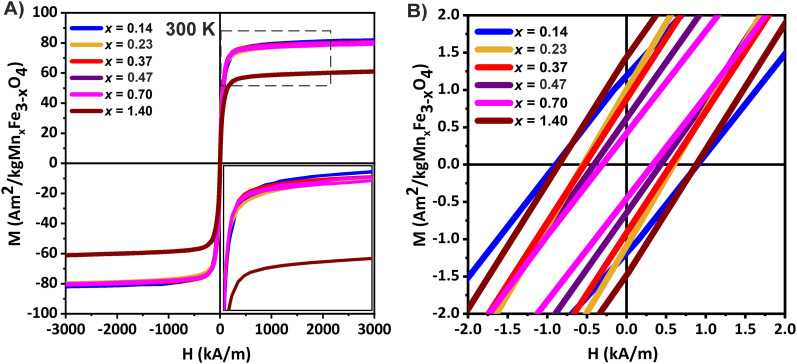
(A) Static magnetization cycles recorded at 300 K for the MNPs and (B) low field region of the magnetization cycles.

The influence of Mn^2+^ substitution on the static magnetic properties was analyzed by recording the hysteresis curves at 300 K using a superconducting quantum interference device (SQUID), as described in the Experimental section. Magnetization values were normalized to the organic content measured by TGA (Fig. S6 right panel in the ESI†) and all the magnetic parameters are presented in Table S5 of the ESI.† Overall, the MNPs showed a SPM behavior at room temperature with negligible remanence or coercivity (Fig. 3). The transition from the FiM to SPM regime stated by Mössbauer spectroscopy is not evidenced here due to the differences in the time windows for both measurements (∼10^−8^*vs.* 10^2^ s). The magnetization values were nearly constant when the Mn^2+^ content varied between *x*_Empiric_ = 0.14–0.70 (80–82 A m^2^ kg_ferrite_^−1^) while decreased for *x*_Empiric_ = 1.40 (61 A m^2^ kg_ferrite_^−1^) (Fig. 3A). Our results are in agreement with the work reported by Li *et al*., where a series of Mn_*x*_Fe_3−*x*_O_4_ MNPs with *x* = 0.11 up to 0.49, and similar particle and crystal sizes (∼ 8.0 nm) showed very similar *M*_S_ values and blocking temperatures despite the differences in composition.^12^ It is worth noting that the expected trend of net magnetic moment improvement as a function of Mn^2+^ content is only valid for normal spinel (*i* = 0), with *i* being the inversion parameter.^56–58^ However, the disruption of this trend has been frequently reported due to the presence of crystal defects promoted by the presence of a secondary phase such as wustite,^2,3,32^ or the oxidation of Mn^2+^ ions to Mn^3+^ and its preference for octahedral sites.^59^

The enrichment of Mn^2+^ cations in the outer part of the MNPs (see Fig. 2D) could promote a configuration where the spins close to the surface are canted and barely contribute to the *M*_S_ in the static regime, explaining the unalterable *M*_S_ values with increasing Mn^2+^ content. The heterogeneous allocation of Mn^2+^ cations and the level of Mn^2+^ substitution could play a major role in reducing the average *H*_C_ and *M*_R_. In fact, even when *M*_S_ values remained nearly constant across all samples, a slight decrease in the coercivity value (*H*_C_) was observed while increasing the Mn^2+^ content until *x*_Empiric_ = 0.70; beyond this point, however, an opposite effect was seen for *x*_Empiric_ = 1.40. The reduction of *H*_C_*via* increased Mn^2+^ substitution ultimately led to a decrease in the effective magnetic anisotropy (*K*_eff_)^60^ as also evidenced with Mössbauer measurement, indicating a transition of the MNPs towards soft magnetic behavior (Fig. 3B). An exception is the sample with the highest Mn^2+^ content (*x*_Empiric_ = 1.40), where the presence of structural defects, associated with the concomitance of a reduced phase and Mn^3+^ fraction, effectively impacts their magnetic properties.

Similar results were recently reported for cobalt ferrite Co_*x*_Fe_3–*x*_O_4_ type MNPs, where increasing the cobalt content had little effect on *M*_S_ across samples, but led to a significant increase of *K*_eff_.^61^ In summary, considering that all samples exhibited similar average particle and crystallite sizes, and morphologies, the tuneable *K*_eff_, the preference of Mn^2+^ for tetrahedral sites and the heterogeneous allocations of cations could be the key properties influencing the ultimate magnetic properties.^56^

### 3.5. Mn^2+^ content changes after PMAO polymer coating: influence on the structural and magnetic properties

**Fig. 4:**
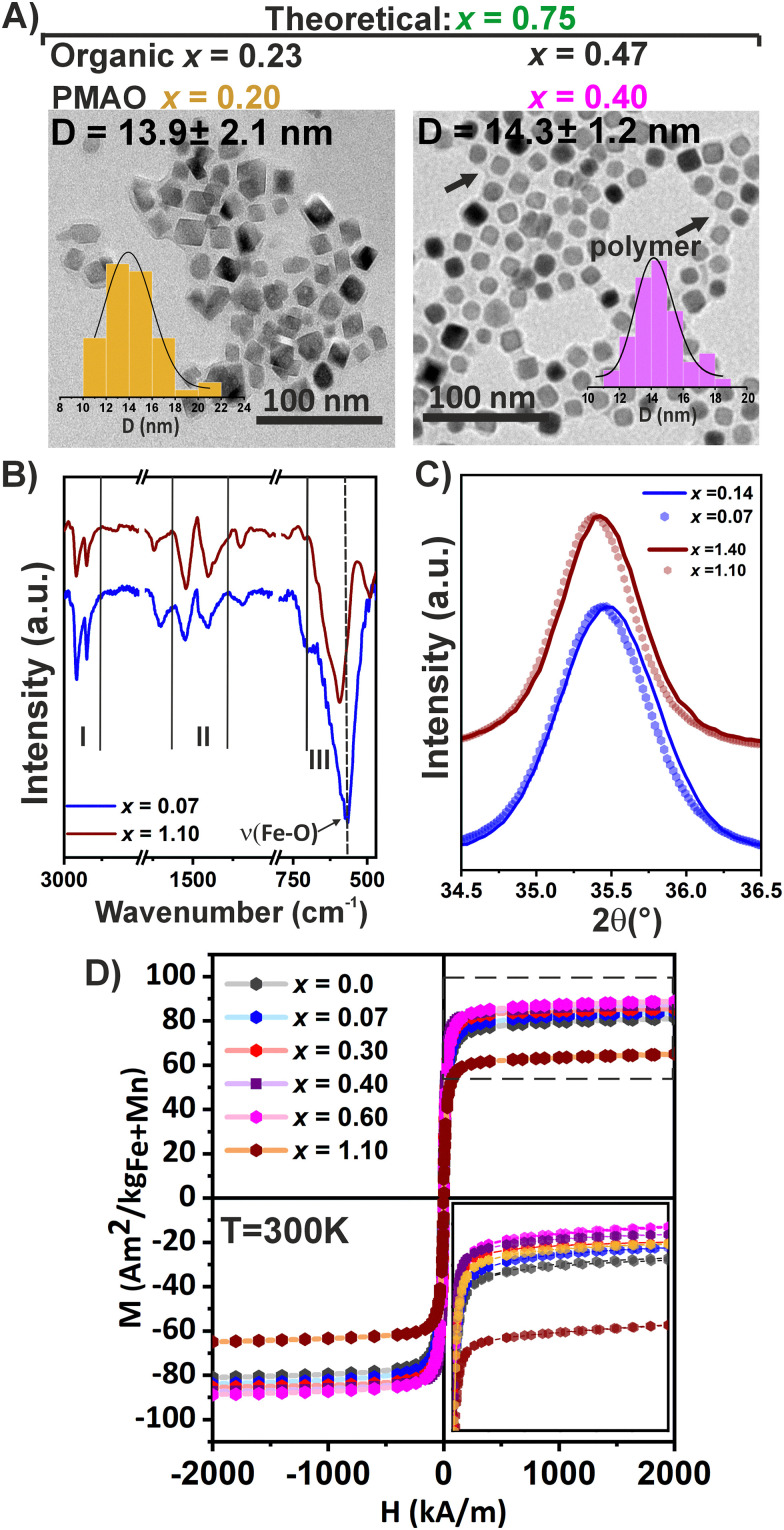
Influence of the PMAO coating on the structural and magnetic properties of Mn_*x*_Fe_3−*x*_O_4_ MNPs. (A) TEM images of selected Mn_*x*_@PMAO samples and their corresponding histogram size distribution. Theoretical and empirical *x* values obtained in organic and aqueous media are shown. (B) FT-IR spectra of Mn_*x*_@PMAO with *x*_Empiric_ = 0.07 and 1.10, respectively. (C) Comparison between XRD patterns with initial composition (organic) of *x*_Empiric_ = 0.14, and 1.40, and after water transference *x*_Empiric_ = 0.07, and 1.10, respectively. (D) Magnetization cycles of Mn_*x*_@PMAO series (*x*_Empiric_ = 0.07 up to 1.10) compared with Fe_3_O_4_ (*x* = 0.0) obtained using the same synthetic procedure at 300 K.

The Mn_*x*_Fe_3−*x*_O_4_ MNPs were prepared in organic solvents where they remained stabilized by an OA coating, so a subsequent transfer to the aqueous phase is required for biomedical applications. To do that, we employed a well-established polymer coating methodology, using an amphiphilic polymer, poly(maleic anhydride-*alt*-1-octadecene) (PMAO), that intercalates with the OA chains.^18^ All MNP series were transferred to water using the same protocol, rendering MNPs stable in aqueous media for a long period of time. Table 2 summarizes the characterization of these MNPs coated with PMAO (Mn_*x*_@PMAO) in water. As expected, the transference into water did not affect the shape and core size of the final MNPs, as can be seen from the TEM image (Fig. 4A), and the hydrodynamic diameter measured by DLS was similar for all MNPs (Table 2). This indicates a similar transfer to water for all the MNPs regardless of the composition, allowing further comparison between the systems. The successful coating of the MNPs was additionally confirmed by TGA (Table 2), as an increase of the organic content between 10 and 24% when compared with samples only coated with OA; this is in agreement with other reported PMAO-coated MNPs.^18,62^

**Table 2:** Summary of the physico-chemical properties of Mn_*x*_@PMAO MNPs. Hydrodynamic radius (*D*_DLS_) is presented in terms of intensity distribution

Samples	Empirical *x* value before PMAO	Empirical *x* value after PMAO	Measured formula	*D*_TEM_ (nm ± σ)	*D*_DLS_ (nm ± SD)	*Z* potential (mV ± SD)	Organic content (%)
Mn_*x*_@PMAO	0.14	0.07	Mn_0.07_Fe_2.93_O_4_	12.9 ± 1.9	51.1 ± 1.6	−38.1 ± 7.2	39.9
	0.23	0.20	Mn_0.20_Fe_2.80_O_4_	13.9 ± 2.1	65.5 ± 0.7	−37.7 ± 6.1	27.3
	0.37	0.30	Mn_0.30_Fe_2.70_O_4_	14.6 ± 2.3	66.5 ± 1.9	−31.2 ± 4.8	23.6
	0.47	0.40	Mn_0.40_Fe_2.60_O_4_	14.3 ± 1.2	66.6 ± 0.4	−39.6 ± 8.2	32.0
	0.70	0.60	Mn_0.60_Fe_2.40_O_4_	14.4 ± 1.8	69.4 ± 2.3	−34.3 ± 6.8	28.4
	1.40	1.10	Mn_1.10_Fe_1.90_O_4_	13.6 ± 1.3	66.5 ± 1.8	−32.3 ± 4.2	34.6

Surface modification can play a major role in the redistribution of cations, ultimately modifying the final composition of the spinel structure and its functionality.^63,64^ To study the influence of the PMAO coating on the composition and, thus, on the structural and magnetic properties, the samples were characterized after coating by ICP-OES, FT-IR and XRD. From the ICP-OES results, we confirmed that Mn^2+^ ions leached out from the MNPs after PMAO coating (Table 2), as previously reported after DMSA or dopamine coatings.^16^ Mn^2+^ losses ranged from 13.0 to 21.4% of the initially incorporated amount across samples, except for the sample containing the lowest content of Mn^2+^, which exhibited a loss of nearly 50% of its Mn^2+^ ions. This trend aligns with the XPS data (Fig. 2D).

The stability of cations occupying surface sites also depends on their interaction with the surface ligand and/or the solvent.^56^Fig. 4B shows the FT-IR spectra of the selected MNPs with the lowest (*x*_Empiric_ = 0.07) and the highest Mn^2+^ content (*x*_Empiric_ = 1.10) after PMAO coating. In region I, the signals of *ν*(C–H) stretching mode belonging to the OA anchored to the surface of MNPs dominate, being more intense for the sample with the highest percentage of OA (*x*_Empiric_ = 0.07), as also confirmed by TGA. Next, region II is dominated by two broad bands that correspond to *ν*_as_(COO^−^) and *ν*_s_(COO^−^) modes of metal carboxylates, indicating the successful polymer coating. The intense band that appears in region III is characteristic of lattice Fe–O vibrations in spinel ferrites. Interestingly, as the Mn^2+^ content increased, this band showed a left shift, indicating the modification of Fe–O bond lengths and the rearrangement of the oxygen sublattice to accommodate different cation distributions among the octahedral and tetrahedral sites, which was affected by PMAO coating.^56^ In agreement with this, a gradual shift in the mean peak (311) position can also be seen by comparing the XRD patterns of samples before and after polymer coating, while the spinel ferrite structure was maintained (Fig. 4C).

The effect of polymer coating on the magnetic properties was evaluated at 5 K and 300 K, with results summarized in Table 3. In agreement with initial oleic acid coated MNP behavior, both the *H*_C_ and the *M*_R_ decreased to near zero values at RT, indicating that the SPM regime was preserved after surface modification. The *M*_S_ values were slightly higher (85.0–89.0 A m^2^ kg_ferrite_^−1^) than those of initial MNPs (see Section 3.4 and Fig. 3) or Fe_3_O_4_ MNPs (82 A m^2^ kg^−1^) obtained under the same synthetic procedure at 300 K (Fig. 4D). Changes in the magnetic properties after surface modification have been previously reported when using different polymers such as triethyleneglycol (TEG) and polyethyleneglycol (PEG), attributed to the reduction of the spin canting effect.^65,66^ However, this does not fully apply in our case, as the PMAO polymer is not directly coordinated to the iron ions but rather intercalated in the OA chains; nevertheless, the PMAO coating could be associated with ion leaching and subsequent cation's redistribution on the MNP surface,^60,67^ which is relevant for applications dependent on the surface properties such as AC hysteresis and relaxometry.^63^

**Table 3:** Magnetic parameters of Mn_*x*_@PMAO samples at 300 K and 5 K

Samples Mn_*x*_@PMAO	300 K			5 K				
	*M*_S_ (A m^2^ kg^−1^)	*H*_C_ (kA m^−1^)	*M*_R_ (A m^2^ kg^−1^)	*M*_S_ (A m^2^ kg^−1^)	*H*_C_ (kA m^−1^)	*M*_R_ (A m^2^ kg^−1^)	*H*_K_ a (kA m^−1^)	*K*_eff_ (10^5^ J m^−3^)
Mn_0.07_Fe_2.93_O_4_	85	2.6	16	100	22.9	40	100	3.9
Mn_0.30_Fe_2.70_O_4_	86	2.6	16	101	20.5	39	80	3.2
Mn_0.40_Fe_2.60_O_4_	87	2.6	17	102	19.4	37	72	2.9
Mn_0.60_Fe_2.40_O_4_	89	2.6	17	107	16.6	44	64	2.7
Mn_1.10_Fe_1.60_O_4_	66	2.6	5	85	24.1	18	100	3.3
Fe_3_O_4_	82	2.6	12	92	22.9	34	80	2.9

a Determined as the field at which the magnetization of the magnetized and the demagnetized branches differs in 3% of *M*_S_.^60^

The effective anisotropy constant (*K*_eff_) values of the different MNP@PMAO samples were estimated from the formula 
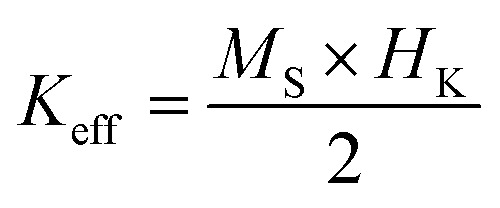
, with *M*_S_ and *H*_K_ being measured at 5 K following the Stoner and Wohlfarth model.^60,68^ The obtained values (Table 3) confirmed a decrease in *K*_eff_ from 3.9 to 2.7 × 10^5^ J m^−3^ as the Mn^2+^ content increased up to *x*_Empiric_ = 0.60 and an increase for samples with *x* ≥ 0.60, confirming the direct correlation between *H*_C_ and the Mn^2+^ content. *K*_eff_ values are approximate due to assumptions of full saturation, negligible temperature variation between 0 and 5 K, and limited interparticle interaction effects at high fields. It is noticeable that *K*_eff_ for Mn_0.60_Fe_2.40_O_4_ is close to the values reported for MnFe_2_O_4_ MNPs (1–2 × 10^5^ J m^−3^),^60,69,70^ which highlights the crucial impact of the Mn^2+^ content on the effective anisotropy of the samples.

### 3.6. Tuning heating performance by changing the Mn^2+^ content

The influence of the Mn^2+^ content on the heating capability of the Mn_*x*_@PMAO MNPs was studied by two methods: calorimetry and AC magnetometry. Low- and high-frequency measurements were conducted at magnetic and ion concentration of 2 mg_Fe+Mn_ per mL or 1 mg_Fe+Mn_ per mL, respectively.

#### 3.6.1. Calorimetric measurements

**Fig. 5:**
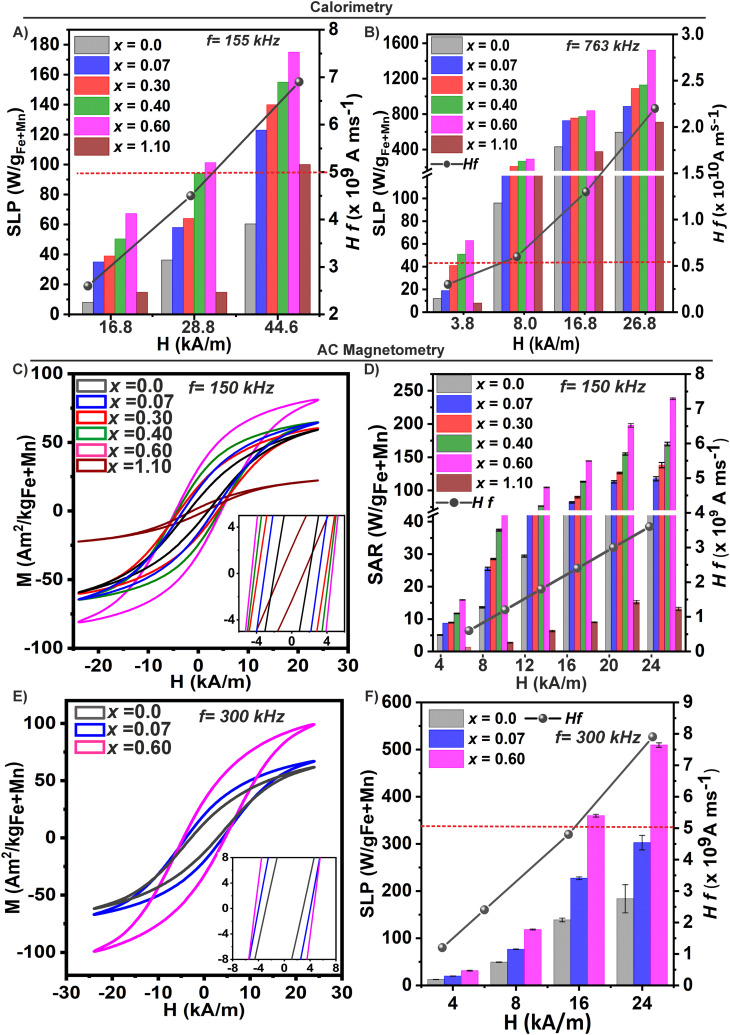
Effect of the Mn^2+^ content on the heating efficiency of Mn_*x*_Fe_3−*x*_O_4_ MNPs. SLP values obtained by the calorimetric method for Mn_*x*_Fe_3−*x*_O_4_ MNPs with fixed frequencies of (A) *f* = 155 kHz and (B) *f* = 763 kHz under field intensity (*H*) ranging from 3.8 up to 44.6 kA m^−1^. (C) Dynamic magnetization curves obtained at 150 kHz and 24 kA m^−1^ for samples with *x*_Empiric_ = 0.0, 0.07, 0.30, 0.40, 0.60, and 1.10. Inset: Magnification of the low field region. (D) SAR values of the corresponding samples (with *x*_Empiric_ = 0.0 up to 1.10). (E) Dynamic magnetization curves obtained at 300 kHz and 24 kA m^−1^ of samples with *x*_Empiric_ = 0.07 and 0.70, respectively, compared to samples without Mn^2+^ (*x* = 0.0). Inset: Low field region. (F) SAR value of samples measured in (E). Black dots represent the *Hf* values for each condition tested. Safe clinic *Hf* values are delimited with a red dotted line.

Fig. 5A and B show the heating performance at different magnetic field intensities ranging from *H* = 3.8 up to 44.6 kA m^−1^ under a low (*f* = 155 kHz) and a high frequency (*f* = 763 kHz), respectively. In general, samples with *x*_Empiric_ = 0.07 up to 0.60 showed a better heating performance than the corresponding iron oxide (*x* = 0.0) MNPs with similar sizes and shapes obtained under the same synthetic procedure. As expected for SPM MNPs, the SLP values increased with the intensity of the applied magnetic field and the Mn^2+^ content (until *x*_Empiric_ = 0.60), being always higher under the high-frequency regime (Fig. 5B). However, for samples with *x*_Empiric_ = 1.10, negligible SLP values were obtained under safe clinical conditions (delimited with a red dotted line). The largest SLP value was recorded for the sample with *x*_Empiric_ = 0.60 under both regimes, in agreement with their improved magnetic properties and low magnetic anisotropy confirmed by the previous data. The heating curve dependence of the applied frequency (155, 388, 637, and 763 kHz) under a fixed *H* = 16.8 kA m^−1^ also showed a linear increase (Fig. S7 in the ESI†). One can conclude that as structural defects and the presence of Mn^3+^ species became significant (*x*_Empiric_ = 1.10), a strong deterioration of *M*_S_ values was observed, and therefore, measurable heating dissipation was only possible under high magnetic field intensity exposure (Fig. 5A and B). Several works have reported the detrimental effect of crystal defects (strains, antiphase boundaries, *etc.*) on heat production.^3,71,72^ This parameter is strictly linked to the synthetic protocol used for the preparation of MNPs and needs to be considered in terms of nanoheater optimization for MHT.

The SLP computed values for high and low-frequency regimes appear in Tables S6 and S7 in the ESI.† These SLP values, shown in Fig. 5A and B, were fitted with a power law SLP = *φH*^*n*^, with *φ* being the mass concentration of the MNPs in the colloid, *H* the amplitude of the applied field, and *n* the power exponent (between 1.99 and 1.09) within the linear response theory (LRT).^73,74^ As low-frequency measurements did not produce accurate fitting, values were not considered. However, under a fixed frequency of 763 kHz (Fig. 5B), the quadratic field dependence of the SLP is fulfilled under all the range of magnetic fields tested, and it is independent of the composition (Table S7 in the ESI†). From these data, we can conclude that the variation of the composition (maintaining a similar size and shape) with the amplitude of the magnetic field only affects the regime of dissipation of the MNPs if the incorporation of Mn^2+^ produces structural defects and/or concomitant with different Mn species like Mn^3+^. In contrast, even with a very different Mn^2+^ content but without any impact on the average structural–magnetic properties, the heat dissipation regimen is warranted within the SPM regime. Still, the SLP values can be successfully tuned with the Mn^2+^ increase.

The magnetic relaxation process of MNPs dispersed in liquids results from individual or combination of Néel and Brown relaxation mechanisms.^73,75^ To unveil between the predominance of Néel and Brown relaxation to produce heat over the anisotropy energy barrier, we tuned the viscosity of the medium containing the MNPs. Specifically, our best nanoheater (*x*_Empiric_ = 0.60) was dispersed in water with increasing final amounts (% v/v) of glycerol (2% and 50%, viscosity range up to ∼ 15 mPa s^75^) and measured by calorimetry at a field intensity of 16.8–28.8 kA m^−1^ and *f* = 763 kHz. We observed that the SLP values remained unaltered when the viscosity of the media was changed (see Fig. S8 in the ESI†), suggesting that the Néel relaxation process is the predominant mechanism.^61,76^ This finding aligns with other works reporting that Fe_3_O_4_ MNPs of 16 nm and 14 nm in water and in glycerol, respectively, are needed to maintain the predominance of Néel relaxation mechanism in a broad range of frequencies (100 kHz to 1 MHz).^61,77^ In contrast, for more advanced compositions such as cobalt ferrite, the Néel relaxation mechanism predominates only within a size range of 6–10 nm.^61,78^ In our case, the predominance of Néel relaxation across compositions and in the range of 13–15 nm just by controlling *K*_eff_ is a crucial strategy for the design of soft ferrite MNPs as heat mediators in complex environments such as tumors, where MNPs tend to aggregate, potentially compromising their magnetic properties.^79–83^

#### 3.6.2. AC magnetization measurements

AC magnetic hysteresis loops were measured for MNPs dispersed in solutions with different Mn^2+^ contents by AC magnetometry to obtain SAR values at 150 and 300 kHz and field intensities (*H*_ac_) ranging from 4 to 24 kA m^−1^ (Fig. 5C–E). In general, the evolution of SAR(*H*_ac_) showed similar trends compared with the calorimetric method. As the Mn^2+^ content increased (until *x*_Empiric_ ≤ 0.60), the areas of the cycles increased under both frequencies at a field amplitude of 24 kA m^−1^, while the opposite trend was observed for samples with *x*_Empiric_ = 1.10 (Fig. 5C and E). The evolution of the AC hysteresis loops as a function of *H*_ac_ (4 up to 24 kA m^−1^) for selected samples (*x*_Empiric_ = 0.07, 0.60, and 1.10) is shown in Fig. S9 in the ESI.† At 150 kHz, *x*_Empiric_ = 1.10 displayed low SAR under all amplitudes tested, exhibiting ‘S’-shaped hysteresis loop curve characteristic of ‘hard ferrite-type’ materials,^84^ whereas, for the rest of the samples, the heating efficiency increases quickly depending on the Mn^2+^ content consistent with the ‘soft-ferrite’ type of MNPs (Fig. 5C, D and Tables S8, S9 in the ESI†). Interestingly, field amplitude-dependent SAR appeared to be linear, with values reaching a plateau with field amplitudes above a threshold of ≥20 kA m^−1^ for MNPs (*x*_Empiric_ = 0.0 up to 0.60), indicating a possible saturation (Fig. S10A in the ESI†).

Fig. 5E depicts the AC hysteresis loops at 300 kHz for samples with *x*_Empiric_ = 0.0, 0.07, and 0.60, also showing a dependence on the Mn^2+^ content. The sample with *x*_Empiric_ = 0.60 presented the largest opening of the AC hysteresis loops, and thus, a SAR value of about 510 W g_Fe+Mn_^−1^, whereas Fe_3_O_4_ MNPs in a similar range of sizes showed a smaller area (SAR = 184 W g_Fe+Mn_^−1^) under the same AMF conditions (300 kHz, 24 kA m^−1^) (Fig. 5F and Table S9 in the ESI†). Linear SAR(*H*_ac_) dependence was maintained as in the case of 150 kHz measurement with a plateau deviation with amplitude ≥20 kA m^−1^ (Fig. S10B in the ESI†).

Aiming to compare both methods, we selected very near low frequencies, 155 kHz for the calorimetric method and 150 kHz for AC magnetometry at a fixed *H* of 16.8 kA m^−1^. Considering our best nanoheater (*x*_Empiric_ = 0.60), we obtained a SLP = 67.3 W g_Fe+Mn_^−1^ by calorimetry compared with SAR = 144.5 W g_Fe+Mn_^−1^ by magnetometry. These differences could be attributed to the non-adiabatic conditions employed for calorimetry measurements, resulting in lower d*T*/d*t* depending on the thermal exchange of MNP suspension with the surrounding media.^85^ However, if we compare the results depicted in Fig. 5A and D it is evident that the heating capacity tendency as a function of the Mn^2+^ content is maintained in both methods, showing their complementarity.

### 3.7. MNP–cell interaction

**Fig. 6:**
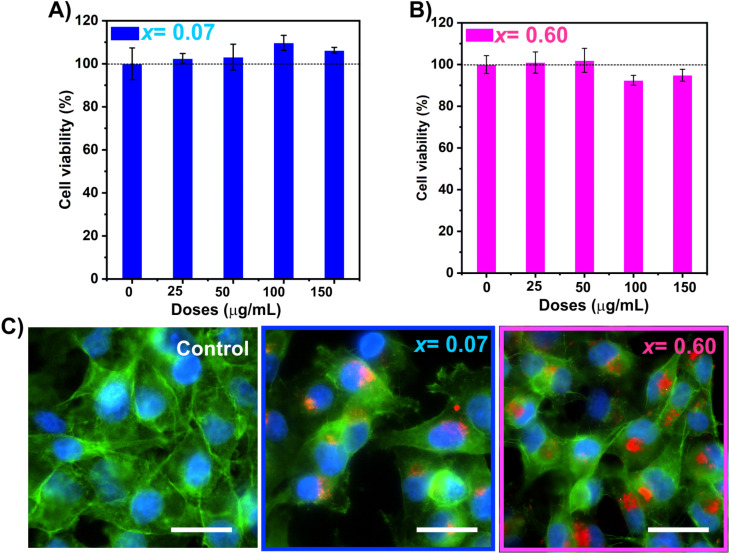
(A) and (B) Viability assay of the MIA PaCa-2 cell line loaded Mn_*x*_Fe_3−*x*_O_4_ MNPs, with *x* = 0.07 and 0.60, respectively. (C) Fluorescence images taken after 24 h of incubation with 100 μg mL^−1^ of TAMRA-labelled MNPs in MIA PaCa-2 cells. Nuclei (blue), F-actin (green) and MNPs (red). Scale bar = 20 μm.

Recently, we reported that tuning the Mn^2+^ composition is crucial to modulate the amount of reactive oxygen species (ROS) produced under MHT to enhance tissue regeneration in the invertebrate model organism *Hydra vulgaris*.^86^ To study the future potential of Mn_*x*_Fe_3−*x*_O_4_ MNPs for cancer treatment, we selected two MNPs with different heating capacities (*x*_Empiric_ = 0.07 and 0.60). To confer improved stability in biological media and target tumoral cells, the samples were functionalized with glucose molecules.^19^ The cytotoxicity of Mn_0.07_Fe_2.93_O_4_ and Mn_0.60_Fe_2.40_O_4_ MNPs functionalized with glucose at different concentrations (ranging from 0 to 150 μg mL^−1^) was evaluated by MTT assay, a well-known colorimetric method used to assess cell metabolic activity as an indicator of cell viability. Cell viability of the MIA PaCa-2 cell line was greater than 90% for both systems (*x*_Empiric_ = 0.07 and 0.60, respectively) after 24 h of treatment, suggesting the absence of cell cytotoxicity and the biocompatibility of MNPs up to 150 μg mL^−1^ (Fig. 6A and B). These results are consistent with previously published data where low levels of toxicity are reported in different cell lines for manganese iron oxide nanoparticles.^16,87,88^ Similarly, these results mirror those obtained *in vivo* in *Hydra vulgaris*, where none of the both MNPs resulted toxic.^86^

Then, we assessed the internalization of Mn_*x*_Fe_3−*x*_O_4_ in the MIA PaCa-2 cell line by fluorescence microscopy. As can be seen in Fig. 6C, large amounts of both Mn_*x*_Fe_3−*x*_O_4_ MNPs appeared internalized inside the cells as red spots in comparison with the control (cells without MNPs), suggesting high cellular uptake after 24 h of treatment. This result is important for applications like classical magnetic hyperthermia where high internalization of iron is required to achieve therapeutic heat. To confirm that, the intracellular iron content was measured by ICP-OES. After 24 h of treatment, the amount of intracellular Fe was 8 and 15 pg per cell for Mn_0.07_Fe_2.93_O_4_ and Mn_0.60_Fe_2.40_O_4_ MNPs, respectively. The different uptake between samples could be attributed to a different amount of glucose functionalized on the MNP surface, or to a diverse stability in biological media. Further studies to elucidate the dependence between MNPs composition and cellular internalization are still needed.

## 4. Conclusions

Besides the shape and size, the composition of MNPs is a critical parameter to tune heating efficiency. In this study, we investigated the effects of the manganese content on the structural, magnetic and heating response of Mn_*x*_Fe_3–*x*_O_4_ MNPs before and after water transference. Our study reveals a compositional gradient in the Mn/Fe atomic ratios across the MNPs, with the surface layers being enriched in Mn^2+^ relative to the deeper core regions. This heterogeneity becomes more pronounced as the Mn^2+^ substitution level decreases. The data suggest that Mn ions primarily incorporate into the ferrite structure starting from the outermost layers, gradually extending toward the interior of the nanoparticles. The surface of the nanoparticles, enriched with Mn^2+^, likely exhibits a SPM relaxation, while the inner core remains predominantly FiM. We have successfully tuned the *K*_eff_ by varying the Mn^2+^ content in the samples with *x*_Empiric_ ≤ 0.70 while keeping the *M*_S_ and crystallite sizes relatively constant. In contrast, a higher Mn^2+^ content (*x*_Empiric_ = 1.40) exhibited additional secondary crystalline phase, strain defects, and the presence of Mn^3+^ species, affecting the *M*_S_ values although the final crystallite size also remained unalterable. Water transference by PMAO coating caused ion leaching that clearly changed the initial composition of the MNPs, but average static magnetic properties were maintained. The heating efficiency was effectively tuned by changing the Mn^2+^ content, being the ferrite with *x*_Empiric_ = 0.60 the one with the optimal Mn^2+^ concentration and anisotropy that maximize SLP. The Néel relaxation process was the predominant mechanism to produce heat for our set of MNPs. Importantly, cell viability remained greater than 90% for the Mn^2+^ tested concentration, suggesting that this system is safe after 24 hours of treatment. Essentially, our study highlights the importance of fine-tuning these properties and presents a valuable framework for advancing magnetic hyperthermia through targeted nanoparticle design. Moreover, the ability to control magnetic anisotropy and heating performance through the Mn^2+^ content highlights the potential of these nanoparticles for biomedical applications, including localized *in vivo* hyperthermia treatments, as recently demonstrated in *Hydra vulgaris*.^86^

## Author contributions

O. F. O.: investigation, XPS measurement and analysis, data curation, and writing – original draft. G. T.: investigation, biological experiment, data curation, and writing – original draft. F. J. T.: resources and review and editing. J. G. O.: investigation and review and editing. J. R.: Mossbauer measurement and analysis and writing – original draft. M. M.: conceptualization, funding acquisition, project administration, supervision and review and editing. S. DS. F.: conceptualization, investigation, data curation, formal analysis, writing – original draft and review and editing.

## Conflicts of interest

There are no conflicts to declare.

## Supplementary Material

NH-010-D5NH00254K-s001

## Data Availability

The data supporting this article have been included as part of the ESI.†
