# Supplementary material for: Unraveling the Mn2+ substitution effect on the anisotropy control and magnetic hyperthermia of MnxFe3−xO4 nanoparticles
Source: Nanoscale Horiz. 2025 Jul 23;10(10):2486–503. doi: 10.1039/d5nh00254k (PMC12319668; doi:10.1039/d5nh00254k)
Supplement: NH-010-D5NH00254K-s001 [file NH-010-D5NH00254K-s001.pdf]

Supplementary Information (SI) for Nanoscale Horizons.
This journal is © The Royal Society of Chemistry 2025

Unraveling the Mn^{2+} Substitution Effect on the Anisotropy Control and Magnetic Hyperthermia of $Mn_xFe_{3-x}O_4$ Nanoparticles

Oscar F. Odio,[a] *Giuseppina Tommasini,*[b] *F. J. Teran,*[c,d] *Jesus G. Ovejero,*[e] *Javier Rubín,*[f] *María Moros*,*[b,g] *Susel Del Sol-Fernández**[b,h]

[a]CONACyT-Instituto Politécnico Nacional, Centro de Investigación en Ciencia Aplicada y Tecnología Avanzada, 11500 Ciudad de México, México

[b]Instituto de Nanociencia y Materiales de Aragón, INMA (CSIC-Universidad de Zaragoza), C/ Pedro Cerbuna 12, 50009, Zaragoza, Spain.

[c]iMdea Nanociencia, Campus Universitario de Cantoblanco, 28049 Madrid, Spain

[d]Unidad de Nanomateriales Avanzados, iMdea Nanociencia, Unidad Asociada al CSIC.

[e]Instituto de Ciencia de Materiales de Madrid, ICMM (CSIC), Sor Juana Inés de la Cruz 3, 28049 Madrid, Spain.

[f]Dept. Materials Science and Metallurgy, Escuela de Ingeniería y Arquitectura (EINA), Universidad de Zaragoza, María de Luna 3, 50018 Zaragoza, Spain.

[g]Centro de Investigación Biomédica en Red de Bioingeniería, Biomateriales y Nanomedicina (CIBER-BBN), Spain.

[h] Present adress: Instituto de Ciencias Físicas, Universidad Nacional Autónoma de México, Av. Universidad s/n, Col. Chamilpa, Cuernavaca, Morelos 62210, México.

Corresponding authors: *Susel Del Sol-Fernández (sdelsol@icf.unam.mx) and María Moros (m.moros@csic.es)*

Table S1. Experimental conditions employed for the synthesis of the two series of MNPs by one-step thermal decomposition method.

Parameters	A Series (150 mL)			B Series (50 mL)			
n(Fe (acac)$_3$) (mmol)	13	11.25	10	4.33	3.75	3.33	2.5
n(Mn(acac)$_2$) (mmol)	2	3.75	5	0.67	1.25	1.67	2.5
initial Fe/Mn ratio	6.5	3	2	6.5	3	2	1
n(OA) (mmol)	40			15			
OA/Fe-Mn ratio	2.6			3.0			
n(HDD) (mmol)	30			10			
V(BE) (mL)	150			50			

Table S2. Experimental conditions employed for the synthesis of Mn ferrite with the same initial Fe/Mn ratio but, increasing surfactant/metallic precursor ratio.

Parameters	2.6	3
n(Fe (acac)$_3$) (mmol)	4.33	
n(Mn(acac)$_2$) (mmol)	0.67	
initial Fe/Mn ratio	6.5	
n(OA) (mmol)	13	15
OA/Fe-Mn ratio	2.6	3.0
n(HDD) (mmol)	10	
V(BE) (mL)	50	

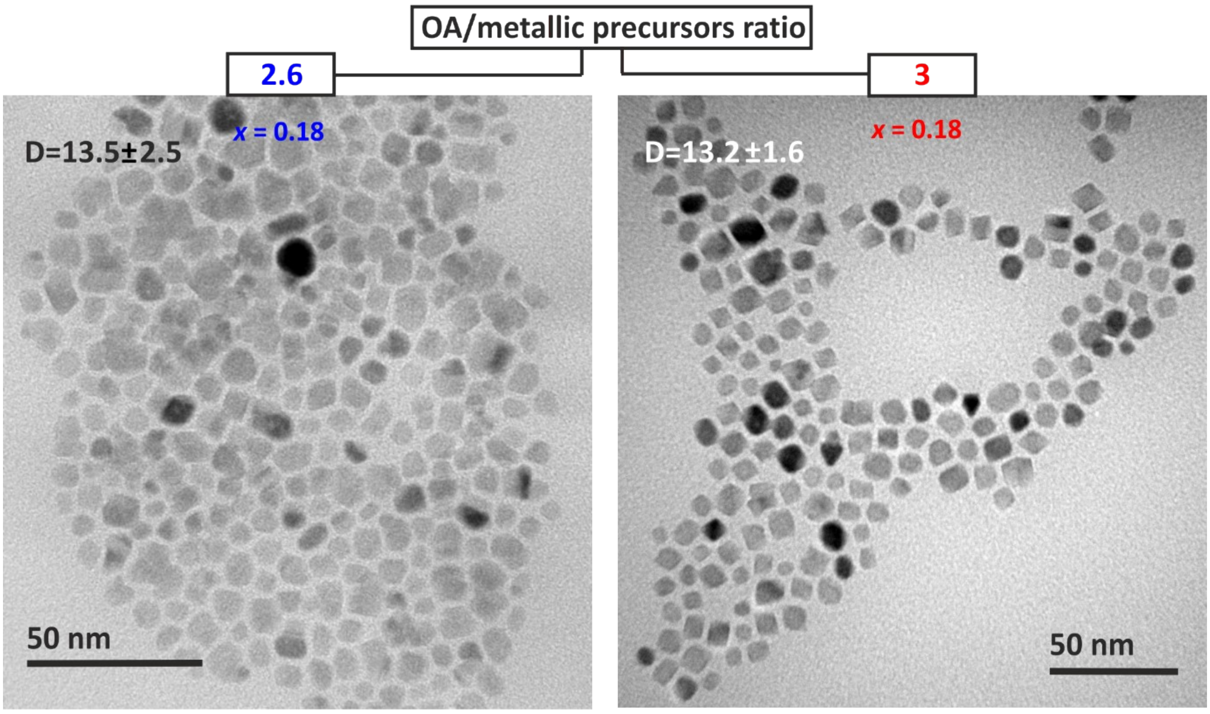

Figure S1. Comparative TEM images of $Mn_xFe_{3-x}O_4$ MNPs obtained under the same synthetic parameters but slightly varying the ratio of OA/Fe-Mn from 2.6 to 3.

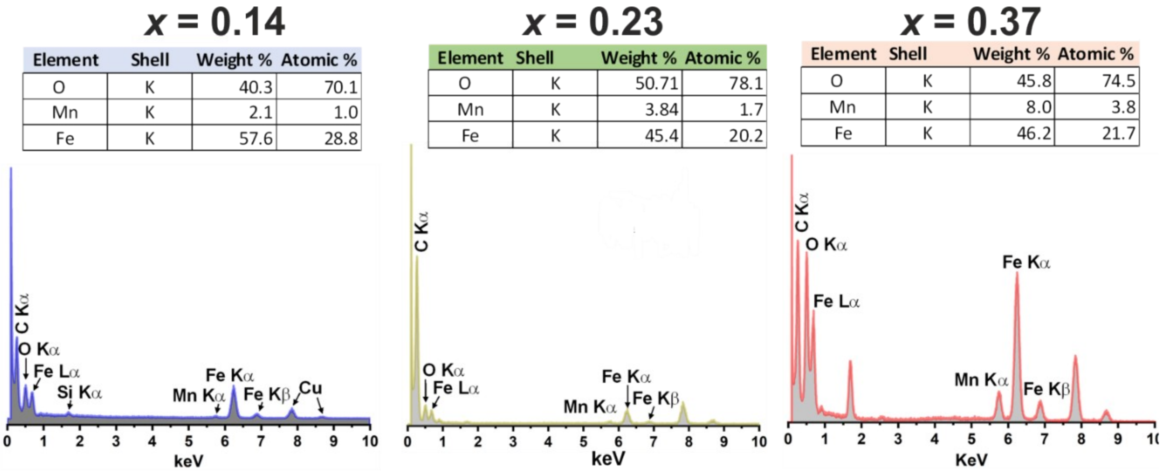

Figure S2. Representative EDX of samples with x = 0.14, 0.23, and 0.37 in organic media (A Series).

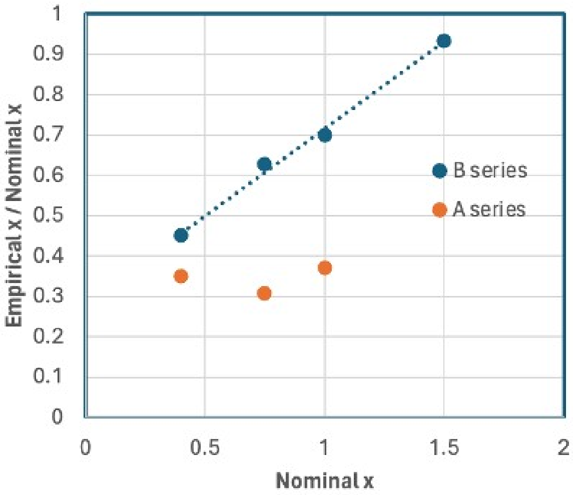

Figure S3. Relation between empirical and nominal x for A series (150mL) and B series (50 mL) of solvent

Table S3. Lattice strain of the $Mn_xFe_{3-x}O_4$@OA MNPs obtained by Williamson-Hall (W-H) method.

Samples	Strain (ε) x 10^{-3}
x = 0.14	0.20
x = 0.23	0.90
x = 0.37	1.26
x = 0.47	1.30
x = 0.70	1.60
x = 1.40	2.12

Section 1. Curve fitting of the high-resolution spectra of Mn 3s and Fe 3s signals and proper quantification of Mn/Fe ratios

The analysis of Mn 3s and Fe 3s region is challenging for two reasons: **1)** the strong overlapping of both Mn 3s and Fe 3s core shells, the last being the significant contribution in doped ferrites samples; **2)** the intrinsic low sensitivity of these core shells due to low values of the corresponding x-ray photoelectron cross sections, thus yielding spectra with poor signal-to-noise ratios. Despite these shortcomings, the 3s region has been widely used for estimating these cations' valence and bond nature, especially for Mn compounds (see references in the manuscript).

The 3s region of transition metals with incomplete d shells comprises mainly a doublet with additional spectral structures more or less pronounced as function of the metal ion and/or the ligand, whose origin and nature have been largely discussed in the past decades (see references in the manuscript). The general consensus states that the main doublet are primarily due to the multiplet splitting of the ionic final-state configuration $3s^1 3d^n$ arising from the exchange interaction between the remaining 3s core-electron and the unpaired electrons in the valence 3d shell of the transition metals; this exchange interaction gives rise to a high spin (lower energy) and low spin (higher energy) final-state configurations, where the spin magnetic moments of the 3s and 3d shells are coupled either parallel or anti-parallel. However, the resulting energy difference (ΔBE) is modulated by other two final-state effects: configuration interaction (CI) effects due to the occurrence of intrashell electron correlation, and inter-atomic local screening of the core hole due to charge transfer (CT) from the ligand (L) to the metal 3d orbitals, leading to electron configurations of the type $3s^1 3d^{n+m} L^{-m}$. The mixing of these screened configurations with the unscreened core-hole ones gives rise to satellite peaks and also contributes to the decrease of the splitting energy of the main doublet. Both spectral effects become more pronounced as the compound is more covalent due to the closed-shell screening of the core

hole; besides, they draw intensity predominantly from the low spin configuration, while the high-spin configuration is less affected[1,2]. When the metal-ligand bond is mostly ionic, which, to first approximation, can be considered the be the case for the metal-oxygen bonds forming the ferrite lattice, the occurrence of satellites from CT configurations are less probable, and the spectra is largely dominated by the signals of the main doublet, which retains the unscreened multiplet character.

The results of the fitting procedure for the ferrite nanoparticles are shown in Figure S4 and summarized in Table S4. All spectra were successfully fitted with two sets of doublets and an extra contribution at higher binding energy (BE). The doublet with the lower BE corresponds to Mn cations, and the one with higher BE corresponds to Fe cations. The extra signal of low intensity at higher BEs can be attributed to the combination of a CI satellite from the low-spin multiplet of the Mn ion (predominant in the sample with x = 1.40) and a CT satellite from the low-spin multiplet of the Fe ion (predominant in the samples with smaller x values)[3,4].

Since the relative weight of final-state effects differs from Mn to Fe, and from sample to sample, the quantification of the Mn/Fe atomic ratios derived from the 3s spectral fittings are subjected to significant errors; in fact, we noted that for homogeneous compounds, the Mn/Fe ratio computed by integrating the 2p signals (where there is no overlapping) is systematically below the corresponding ratio computed by the analysis of the 3s signals. To correct this issue, we prepared a series of standard samples by mixing $Fe(ac)_2$ and $Mn(ac)_2$ thoroughly in precise compositions. Then, after the spectral fitting procedure, the relative areas of the high-spin multiplets were plotted against the Mn/Fe atomic ratios from the integration of the high-resolution Mn2p and Fe2p signals. The resulting calibration curve is shown in Figure S5; it is apparent that there is a very good linear correlation in the range of compositions used (despite the inherent uncertainties due to the spectral fits), which covers those of the samples under study. Note that we selected for this analysis the high-spin contribution of the main doublet for

each cation since this multiplet is less affected by the other final-state effects and because it is the most intense component of the doublet, which minimizes the uncertainties introduced by the fitting procedure. The slope of the linear regression fit (forcing the intercept to be zero) is 0.8, which means that this value times the obtained area ratio from a given spectral fit, yields the average Mn/Fe atomic composition along the information depth covered by electrons from the 3s region (*c.a.* 6 nm). Note that the key assumption of this approach is that the standard samples exhibit a high depth homogeneity in order to guarantee that the atomic ratios from the 2p orbitals equal those expected from the 3s region. The values obtained for the samples under study are reported in Table S4.

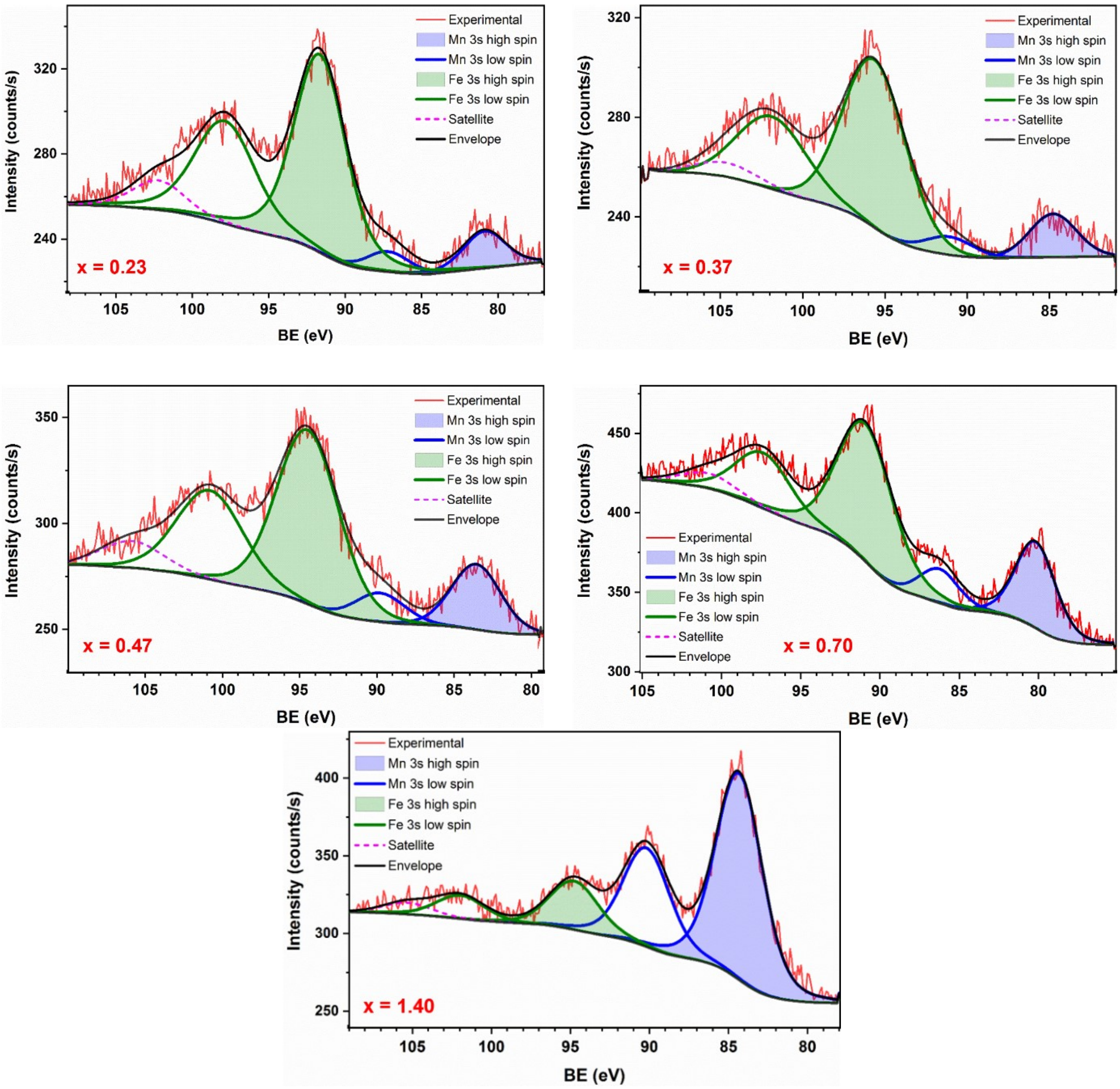

Figure S4. XP high resolution spectra and curve fitting results of the Mn 3s and Fe 3s orbitals corresponding to samples with ferrite stoichiometry, from top to bottom, of x = 0.23, 0.37, 0.47, 0.70 and 1.40.

Table S4. Fitting results of the Mn 3s and Fe 3s high resolution spectral region

Sample	Parameter[a]	Mn 3s	Fe 3s
***x* = 0.23**	ΔBE[b] (eV)	6.3 ± 0.2	6.22 ± 0.08
	FWHM[c] (eV)	3.30 ± 0.04	4.00 ± 0.04
	Mn/Fe[d] (at./at.)	0.12 ± 0.03	
***x* = 0.37**	ΔBE[b] (eV)	6.3 ± 0.2	6.23 ± 0.09
	FWHM[c] (eV)	3.4 ± 0.2	4.55 ± 0.05
	Mn/Fe[d] (at./at.)	0.16 ± 0.05	
***x* = 0.47**	ΔBE[b] (eV)	6.2 ± 0.2	6.33 ± 0.08
	FWHM[c] (eV)	3.8 ± 0.1	4.74 ± 0.05
	Mn/Fe[d] (at./at.)	0.24 ± 0.05	
***x* = 0.70**	ΔBE[b] (eV)	6.1 ± 0.1	6.5 ± 0.1

	FWHM[c)] (eV)	3.30 ± 0.04	4.00 ± 0.04
	Mn/Fe[d)] (at./at.)	0.36 ± 0.09	
***x* = 1.40**	ΔBE[b)] (eV)	5.86 ± 0.04	7.2 ± 0.2
	FWHM[c)] (eV)	3.30 ± 0.04	4.00 ± 0.04
	Mn/Fe[d)] (at./at.)	3.2 ± 0.6	

a) Uncertainties are calculated following the reference[5]

b) Binding energy difference between the peak maxima of the doublet

c) Refers to the full width at half maximum for the high-spin contribution of the doublet

d) Refers to the relative atomic percentage calculated from the computed peak areas, as explained above

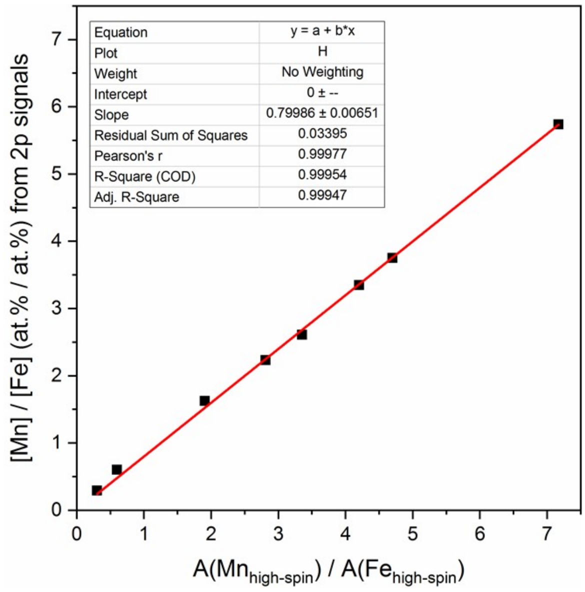

Figure S5. Calibration curve that correlates the Mn/Fe atomic ratio with the area ratio of the high-spin multiplet from the Mn 3s and Fe 3s spectra.

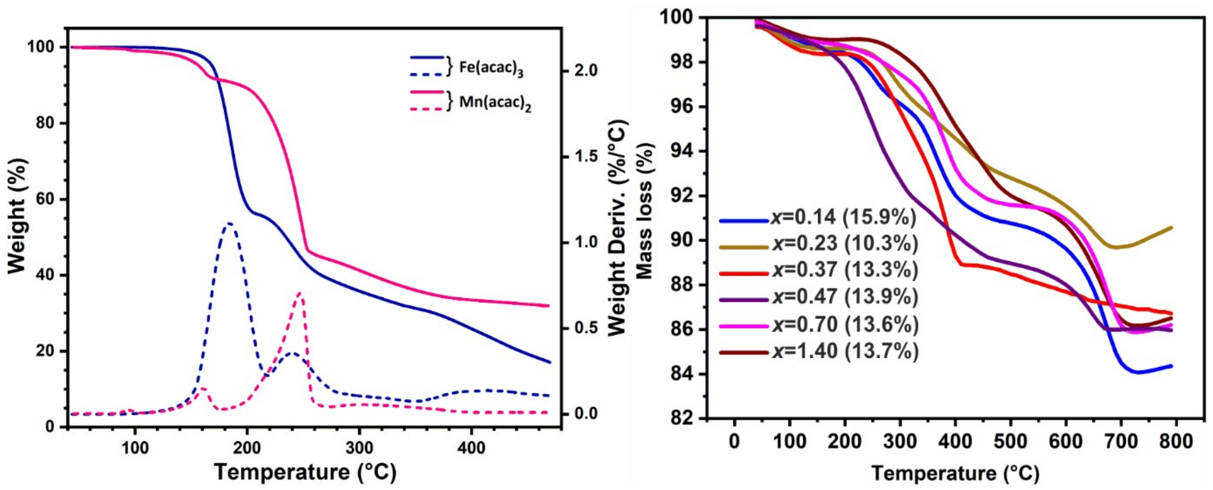

Figure S6. TGA of the metal-organic precursors (continuous and dashed curves correspond, respectively, to weight loss and its temperature derivative) (*left panel*). Organic content of $Mn_xFe_{3-x}O_4$ @OA MNPs, as determined from TGA (*right panel*).

Table S5. Magnetic properties of $Mn_xFe_{3-x}O_4$ @OA MNPs at 300 K.

**Samples**	M_S **(Am²/kgferrite)**	H_C **(Oe)**	M_R **(Am²/kgferrite)**
$Mn_{0.14}Fe_{2.86}O_4$	81.4	11.2	1.2
$Mn_{0.23}Fe_{2.77}O_4$	80.4	6.9	0.9
$Mn_{0.37}Fe_{2.63}O_4$	80.2	7.1	1.1
$Mn_{0.47}Fe_{2.53}O_4$	80.5	5.3	0.7
$Mn_{0.70}Fe_{2.30}O_4$	80.9	4.0	0.40
$Mn_{1.40}Fe_{1.60}O_4$	61	10.9	1.5

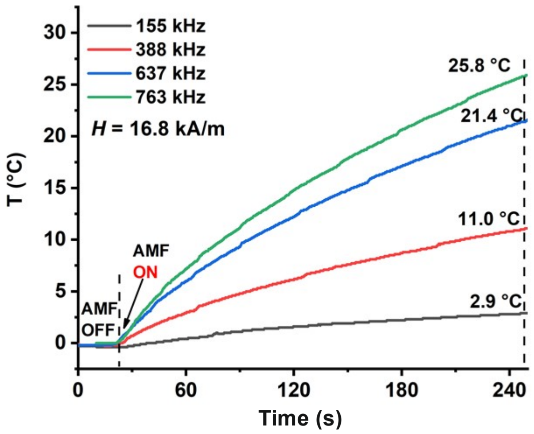

Figure S7. Heating curves for $Mn_{0.60}Fe_{2.40}O_4$ MNP at different frequencies (155 up to 763 kHz) and H = 16.8 kA/m

Table S6. SLP values obtained by **calorimetry** for different compositions of $Mn_xFe_{3-x}O_4$ compared with iron oxide MNP (Fe_3O_4) under a fixed $\boldsymbol{f}$ **= 155 kHz**.

***H* (kA m^{-1})**	Composition Mn_x@PMAO MNPs **SLP (W/g$_{Fe+Mn}$)**					**Fe_3O_4**	***H* x *f* (A ms^{-1})**
	**$Mn_{0.07}Fe_{2.93}O_4$**	**$Mn_{0.30}Fe_{2.70}O_4$**	**$Mn_{0.40}Fe_{2.60}O_4$**	**$Mn_{0.60}Fe_{2.40}O_4$**	**$Mn_{1.10}Fe_{1.60}O_4$**		
16.8	35	39	50	67	15	8	2.6 x 10^9
28.8	58	64	94	101	15	36	4.5 x 10^9
44.6	123	140	155	175	100	60	6.9 x 10^9

Table S7. SLP values obtained by **calorimetry** for different compositions of $Mn_xFe_{3-x}O_4$ compared with iron oxide MNP (Fe_3O_4) under a fixed $\boldsymbol{f}$ **= 763 kHz**. Power exponents (n) that rule the field dependence of the SLP for each sample appear in the last raw (shaded in blue).

***H* (kA m^{-1})**	Composition Mn_x@PMAO MNPs **SLP (W/g$_{Fe+Mn}$)**					**Fe_3O_4**	***H* x *f* (A ms^{-1})**
	**$Mn_{0.07}Fe_{2.93}O_4$**	**$Mn_{0.30}Fe_{2.70}O_4$**	**$Mn_{0.40}Fe_{2.60}O_4$**	**$Mn_{0.60}Fe_{2.40}O_4$**	**$Mn_{1.10}Fe_{1.60}O_4$**		
3.8	19	41	51	63	8	12	2.8 x 10^9
8.0	141	210	269	293	125	96	6.1 x 10^9
16.8	727	756	773	839	377	432	1.3 x 10^{10}
28.8	887	1090	1129	1522	710	596	2.2 x 10^{10}
n	1.5 ± 0.7	1.2 ± 0.3	1.1 ± 0.3	1.3 ± 0.1	1.3 ± 0.1	1.4 ± 0.5	

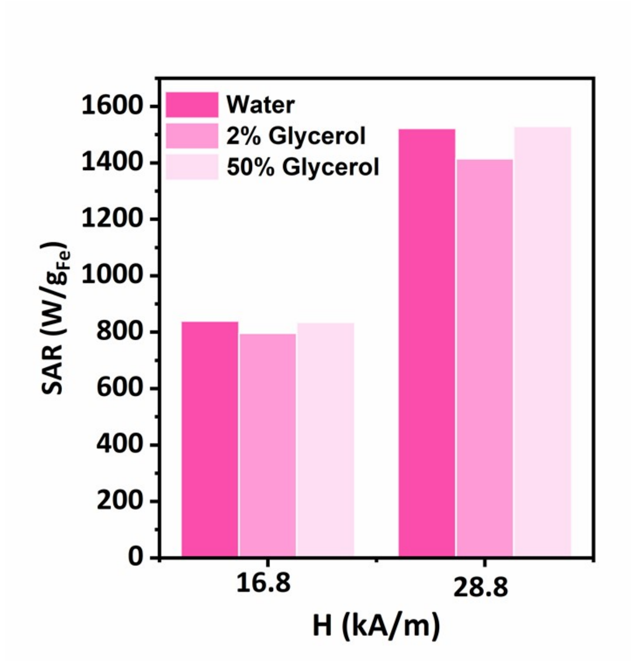

Figure S8. Heating performance dependence of the viscosity of the medium under a fixed frequency of 763 kHz and H between 16.8 up to 28.8 kA/m for sample with $x_{Empiric}$ = 0.60

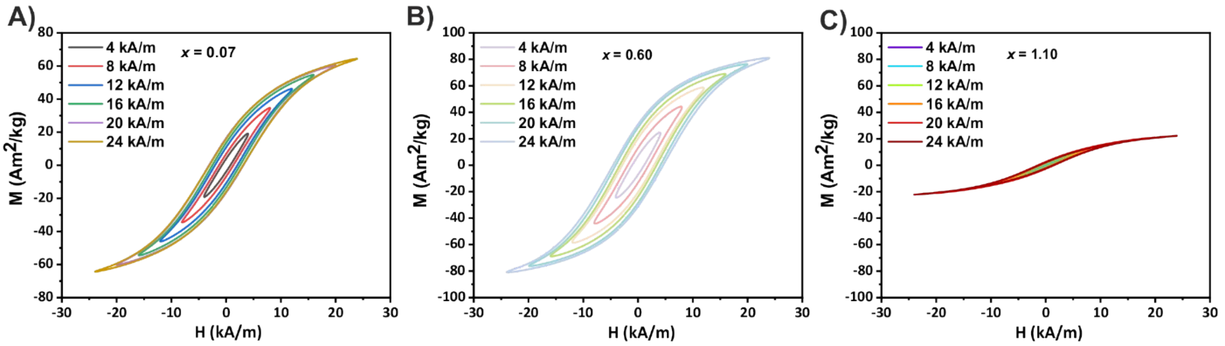

Figure S9. Hysteresis curve as a function of the *Hac* applied for A) x= 0.07, B) x= 0.60, and C) x=1.10 at 150 kHz.

Table S8. SAR values obtained by AC magnetometry for different compositions of $Mn_xFe_{3-x}O_4$ compared with iron oxide MNP (Fe_3O_4) under a fixed f = 150 kHz.

H **(kA m^{-1})**	**Composition Mn$_x$@PMAO MNPs SAR (W/g$_{Fe+Mn}$±SD)**					**Fe_3O_4**	***H* x *f*** **(A ms^{-1})**
	**$Mn_{0.07}Fe_{2.93}O_4$**	**$Mn_{0.30}Fe_{2.70}O_4$**	**$Mn_{0.40}Fe_{2.60}O_4$**	**$Mn_{0.60}Fe_{2.40}O_4$**	**$Mn_{1.10}Fe_{1.60}O_4$**		
4	8.7 ± 0.0	8.9 ± 0.1	11.7 ± 0.1	15.9 ± 0.1	1.3 ± 0.0	5.1± 0.1	6 x 10^8
8	25.5 ± 0.5	28.5 ± 0.2	37.4 ± 0.3	50.5 ± 0.2	2.7 ± 0.1	13.6 ± 0.2	1.2 x 10^9

12	55.3 ± 0.3	60 ± 0.9	76.6 ± 0.2	104.7 ± 0.5	6.3 ± 0.2	29.4 ± 0.3	1.8 x 10^9
16	82 ±1.0	90 ± 1.2	113.2 ± 0.6	144.5 ± 0.2	9.0 ± 0.1	42.1 ± 0.2	2.4 x 10^9
20	113 ± 1.8	126.3 ± 1.1	154.8 ± 1.7	197.9 ± 2.7	15.2 ± 0.5	61.4 ± 1.4	3.0 x 10^9
24	117.5 ± 3.1	138.2 ± 4.0	169.9 ± 2.7	238 ± 1.3	13.1 ± 0.5	64.0 ± 0.7	3.6 x 10^9
n	1.2 ± 0.2	1.6 ± 0.1	1.6 ± 0.1	1.5 ± 0.1	1.2 ± 0.3	1.3 ± 0.2	

Table S9. SAR values obtained by AC magnetometry for different compositions of $Mn_xFe_{3-x}O_4$ compared with iron oxide MNP (Fe_3O_4) under a fixed f = 300 kHz.

***H* (kA m^{-1})**	**Fe_3O_4**	**$Mn_{0.07}Fe_{2.93}O_4$**	**$Mn_{0.60}Fe_{2.40}O_4$**	***Hf* (A ms^{-1})**
4	12.4 ± 0.2	19.7 ± 0.1	31.1 ± 0.7	1.2 x 10^9
8	49.1 ± 0.5	76.6 ± 0.4	118.3 ± 0.9	2.4 x 10^9
16	138.7 ± 3.8	227 ± 2.9	359.8 ± 3.0	4.8 x 10^9
24	183.6 ± 29.8	302.5 ± 15.3	509.5 ± 4.8	1.2 x 10^9
n	1.8± 0.1	1.8± 0.1	1.5± 0.1	

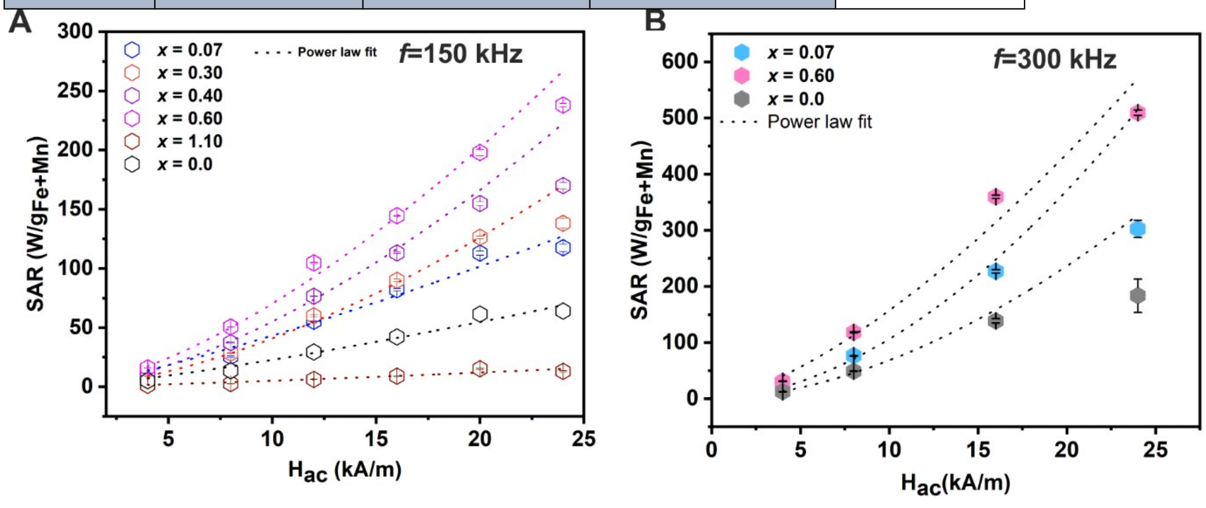

Figure S10. Power law fit (dotted lines) of the SAR dependence with field amplitude (from 4 up to 24 kA/m) under a fixed frequency of **A)**150 kHz and **B)** 300 kHz. The power exponents are labeled as *n* and appear in Table S7-S8.

Table S10. Mössbauer parameters of the spectra recorded at room temperature

Spectral contribution	Parameter[a)]	$x = 0.14$	$x = 0.23$	$x = 0.37$
**S1**	$\delta^{b)}$ (mm/s)	0.35(1)	0.31(3)	0.324(5)
	2ε (mm/s)	-0.04(2)	0.02(2)	-0.01(1)
	B_{HF} (T)	48.5(2)	48.1(2)	48.10(7)
	FWHM (mm/s)	0.43(5)	0.47(7)	0.49(2)
	Relative Area	26.6	12.5	16.3
**S2**	$\delta^{b)}$ (mm/s)	0.31(2)	0.23(2)	0.368(6)
	2ε (mm/s)	0.03(3)	-0.13(4)	-0.02(1)
	B_{HF} (T)	46.5(2)	45.8(3)	46.2(1)
	FWHM (mm/s)	0.4(1)	0.5(1)	0.61(4)

	Relative Area	14.9	13.9	19.1
**S3**	$\delta^{b)}$ (mm/s)	0.46(2)	0.56(5)	0.52(2)
	2ε (mm/s)	0.00(4)	0.23(6)	0.50(4)
	B_{HF} (T)	43.4(3)	44.5(3)	42.2(1)
	FWHM (mm/s)	1.12(8)	0.64(7)	0.58(7)
	Relative Area	52.8	20.1	7.5
**S4**	$\delta^{b)}$ (mm/s)	-	0.45(2)	0.43(1)
	2ε (mm/s)		-0.63(5)	-0.29(3)
	B_{HF} (T)		43.8(2)	42.5(1)
	FWHM (mm/s)		0.4(1)	0.72(6)
	Relative Area		6.1	16.2
**S5**	$\delta^{b)}$ (mm/s)	-	0.44(3)	0.40(2)
	2ε (mm/s)		-0.03(5)	-0.02(4)
	B_{HF} (T)		38.2(3)	36.1(2)
	FWHM (mm/s)		1.1*	1.2*
	Relative Area		19.8	15.2
**S6**	$\delta^{b)}$ (mm/s)	-	0.32(7)	0.36(5)
	2ε (mm/s)		-0.1(1)	-0.26(9)
	B_{HF} (T)		30.9(8)	28.3(4)
	FWHM (mm/s)		1.0*	1.2*
	Relative Area		6.4	7.0
**S*n***	$\delta_0^{b)}$ (mm/s)	-	0.3(1)	0.32(5)
	B_{HF} (T)		15.8(2)	13.0(5)
	ΔB_{HF} (T)		8.3(2)	8.1(5)
	IS-HF$^{c)}$ (mm/S.T)		0.02(2)	0.05(1)
	Linewidth (mm/s)		1.7*	1.0(3)
	Relative. Area		21.3	18.8
**D1**	$\delta^{b)}$ (mm/s)	0.66(7)	-	-
	QS (mm/s)	2.2(2)		
	FWHM (mm/s)	0.8(2)		
	Relative. Area	5.7		
	χ^2	1.035	1.048	1.429

a) Errors in parenthesis
b) Isomer shift relative to α-Fe at room temperature
c) Coupling factor between the isomer shift and the hyperfine field distributions
* Fixed value

References

(1) de Vries A. H.; Hozoi L.; Broer R.; Bagus P. S. Importance of interatomic hole screening in core-level spectroscopy of transition metal oxides: Mn 3s hole states in MnO. *Physical Review B*, **2002**, *66*(3), 035108.

(2) Bagus, P. S.; Ilton, E. S. Effects of covalency on the p-shell photoemission of transition metals: MnO. *Physical Review B*, **2006**, *73*(15), 155110.

(3) Gweon, G. H.; Park, J. G.; Oh, S. J. Final-state screening effect in the 3s photoemission spectra of Mn and Fe insulating compounds. *Physical Review B*, **1993**, *48*(11), 7825-7835.

(4) Sangaletti, L.; Depero, L. E.; Bagus, P. S.; Parmigiani, F. A proper Anderson Hamiltonian treatment of the 3s photoelectron spectra of MnO, FeO, CoO and NiO. *Chemical Physics Letters*, **1995**, *245*(4-5), 463-468.

(5) Evans, S. Estimation of the uncertainties associated with XPS peak intensity determination. *Surface and Interface Analysis*, **1992**, *18*(5), 323-332.
